# p53 Regulates Cell Cycle and MicroRNAs to Promote Differentiation of Human Embryonic Stem Cells

**DOI:** 10.1371/journal.pbio.1001268

**Published:** 2012-02-28

**Authors:** Abhinav K. Jain, Kendra Allton, Michelina Iacovino, Elisabeth Mahen, Robert J. Milczarek, Thomas P. Zwaka, Michael Kyba, Michelle Craig Barton

**Affiliations:** 1 Program in Genes and Development, Center for Stem Cell and Development Biology, Department of Biochemistry and Molecular Biology, The University of Texas MD Anderson Cancer Center, Houston, Texas, United States of America; 2 Lillehei Heart Institute and Department of Pediatrics, University of Minnesota, Minneapolis, Minnesota, United States of America; 3 Center for Cell and Gene Therapy, Baylor College of Medicine, Houston, Texas, United States of America; B.C. Cancer Agency, Canada

## Abstract

Multiple studies show that tumor suppressor p53 is a barrier to dedifferentiation; whether this is strictly due to repression of proliferation remains a subject of debate. Here, we show that p53 plays an active role in promoting differentiation of human embryonic stem cells (hESCs) and opposing self-renewal by regulation of specific target genes and microRNAs. In contrast to mouse embryonic stem cells, p53 in hESCs is maintained at low levels in the nucleus, albeit in a deacetylated, inactive state. In response to retinoic acid, CBP/p300 acetylates p53 at lysine 373, which leads to dissociation from E3-ubiquitin ligases HDM2 and TRIM24. Stabilized p53 binds *CDKN1A* to establish a G_1_ phase of cell cycle without activation of cell death pathways. In parallel, p53 activates expression of *miR-34a* and *miR-145*, which in turn repress stem cell factors OCT4, KLF4, LIN28A, and SOX2 and prevent backsliding to pluripotency. Induction of p53 levels is a key step: RNA-interference-mediated knockdown of p53 delays differentiation, whereas depletion of negative regulators of p53 or ectopic expression of p53 yields spontaneous differentiation of hESCs, independently of retinoic acid. Ectopic expression of p53R175H, a mutated form of p53 that does not bind DNA or regulate transcription, failed to induce differentiation. These studies underscore the importance of a p53-regulated network in determining the human stem cell state.

## Author Summary

Most cell types in an organism are generated from embryonic stem cells (ESCs), which are able to proliferate an unlimited number of times and have the potential to produce any kind of cell of that organism (this ability is called pluripotency). In order to maintain these properties, ESCs have to remain in a proliferate state, which is achieved by the collaboration of several factors. Expressing combinations of these factors in differentiated cells can result in ESC-like qualities; these induced pluripotent stem cells (iPSCs) can then function like ESCs. Previous studies suggested that p53, generally known for its roles in maintaining genomic integrity by regulating cell cycle and cell death pathways, also acts as a barrier to reprogramming adult cells during the creation of iPSCs; whether this is strictly due to repression of proliferation remains a subject of debate. Here, we show that p53 plays a significant role in actively promoting differentiation of human ESCs (hESCs). We find that, prior to differentiation, p53 is expressed at very low levels in hESCs, held in check by two negative regulators, HDM2 and TRIM24, that trigger p53 degradation. Upon induction of differentiation, lysine 373 of p53 is acetylated, and this disrupts the existing interactions with negative regulators, thus allowing stabilization and activation of p53. Active p53 in turn promotes expression of the cell cycle regulator p21 to slow down the hESC cell cycle; cells in the gap (G_1_) phase of the cell cycle accumulate, preventing division. In parallel, p53 activates specific microRNAs, *miR-34a* and *miR-145*, that inhibit the expression of several stem cell factors and prevent differentiated cells from backsliding to pluripotency. Our results highlight a novel function of p53 in determining the human stem cell state.

## Introduction

Embryonic stem cells (ESCs) have an unlimited potential to proliferate (self-renewal) and the ability to generate and differentiate into most cell types (pluripotency) [1],[2]. The undifferentiated ESC state is regulated by a network of transcription factors, e.g., OCT4, SOX2, NANOG, and KLF4, and epigenetic modifiers, which promote expression of ESC-specific genes and suppress differentiation [3]–[7]. Exogenous introduction of transcription factors such as OCT4, SOX2, NANOG, and KLF4 into murine or human adult cells induces pluripotency by reprogramming these cells into induced pluripotent stem cells (iPSCs), which are functionally and phenotypically similar to ESCs [8],[9].

The ability of ESCs to self-renew and maintain pluripotency is linked to the ability of these cells to remain in a proliferative state. ESCs progress through an abbreviated cell cycle, leading to rapid cell division [10]–[12], characterized by a truncated G_1_ phase, elevated expression of G_1_-associated cyclins, active cyclin-dependent kinases (CDKs), and low levels of inhibitory cell cycle proteins p21, p27, and p57 [13]. During differentiation to embryoid bodies, mouse ESCs (mESCs) accumulate in G_1_ and exhibit a cell cycle lengthened from 8–10 h to more than 16 h, as observed in adult cells [14]. In the creation of iPSCs, multiple studies show more efficient reprogramming of cells with dysfunctional ARF/p53 pathways and increased cellular proliferation, shortening of G_1_, and lack of cell cycle checkpoints [15]–[20]. Additional studies identified several small non-coding RNAs that play roles in cell cycle regulation and control of ESC status [21]. MicroRNAs (miRNAs) are small, non-coding RNAs of 21–23 nucleotides in length that regulate gene expression, generally at a post-transcriptional level [22]. Specific miRNAs regulate self-renewal, pluripotency, and mESC stability [23],[24], and several are differentially expressed in human ESCs (hESCs) [25],[26].

Here, we connect both regulatory arms, e.g., cell cycle progression and transcription of miRNAs, to tumor suppressor p53 in regulated differentiation of hESCs. Exposure of hESCs to differentiating conditions signals an acetylation switch to stabilize p53 protein. Activation of p53 elongates the G_1_ phase of the cell cycle by p21 induction, and increases *miR-34a* and *miR-145*, which target specific stem cell factors for repression. These functions of p53 are direct, as ectopically expressed p53 binds these chromatin targets and causes spontaneous differentiation without retinoic acid (RA) addition. The combined effects of p53 not only antagonize self-renewal and pluripotency but also have an active role in promoting differentiation of hESCs.

## Results

### p53 Protein Is Induced during Differentiation of hESCs

**Figure 1:**
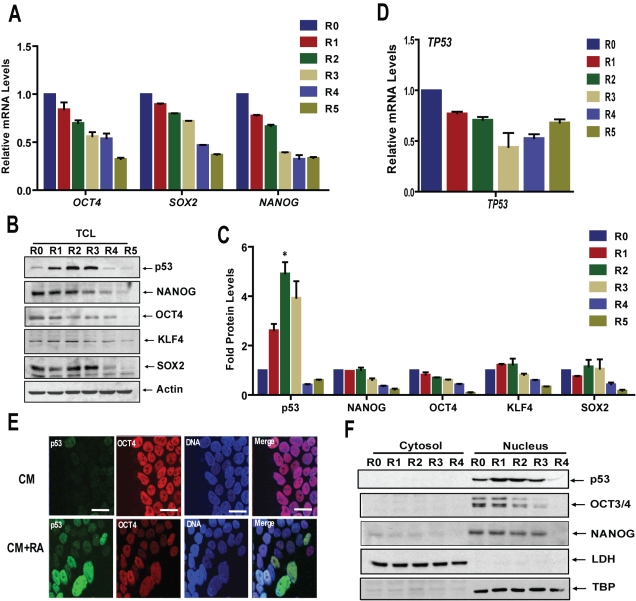
p53 protein is induced during differentiation of hESCs. (A) qRT-PCR. RNA from hESCs treated with RA in medium without FGF for 5 d (R0–R5), were subjected to qRT-PCR assay (data are presented as mean ± SEM). (B and C) Western blot. Lysates (total cell lysate [TCL]) prepared from hESCs cultured as in (A) were analyzed by Western blotting, the blots in (B) were quantitated (C): the average density of three different blots is plotted (*, *p*<0.05). (D) *TP53* qRT-PCR. RNA samples prepared as in (A) were subjected to qRT-PCR assay (mean ± SEM). (E) Immunofluorescence. hESCs in complete CM or treated with RA for 3 d were stained with antibodies against p53 and OCT4, and nuclei were stained with DAPI. Scale bar is 50 µm. (F) p53 nuclear localization. Nuclear extracts prepared from hESCs cultured as in (A) were analyzed by Western blotting. (Also see Figure S1.).

In vitro differentiation of hESCs (WA09 cells), induced by addition of RA and withdrawal of fibroblast growth factor (FGF), is marked by a steady decline in levels of proteins and RNAs associated with pluripotency and self-renewal, e.g., NANOG, OCT4, SOX2, and KLF4, and increased expression of endodermal marker *GATA4*, *AFP*, ectodermal marker *PAX6*, and neural progenitor gene *Nestin* (Figures 1A–1C and S1A), as previously described [27]. In parallel, p53 protein levels increase significantly but transiently (Figure 1B and 1C) without increased *TP53* transcription (Figure 1D). In response to RA, induced p53 is nuclearly localized in differentiating cells (Figures 1E, 1F, and S1B), which are identifiable by the loss of homogeneity and elongated nuclei seen in highly p53-expressing cells (Figure 1E). As one of several examples where hESCs differ from mESCs [28],[29], p53 is expressed at low levels and nuclearly enriched in hESCs prior to induction (Figures 1B, 1E, and S1C). Transient induction of p53 protein levels during RA-mediated differentiation was also observed in WA01 hESCs (Figure S1D and S1E), and in BMP4-mediated differentiation of hESCs (data not shown).

**Figure 2:**
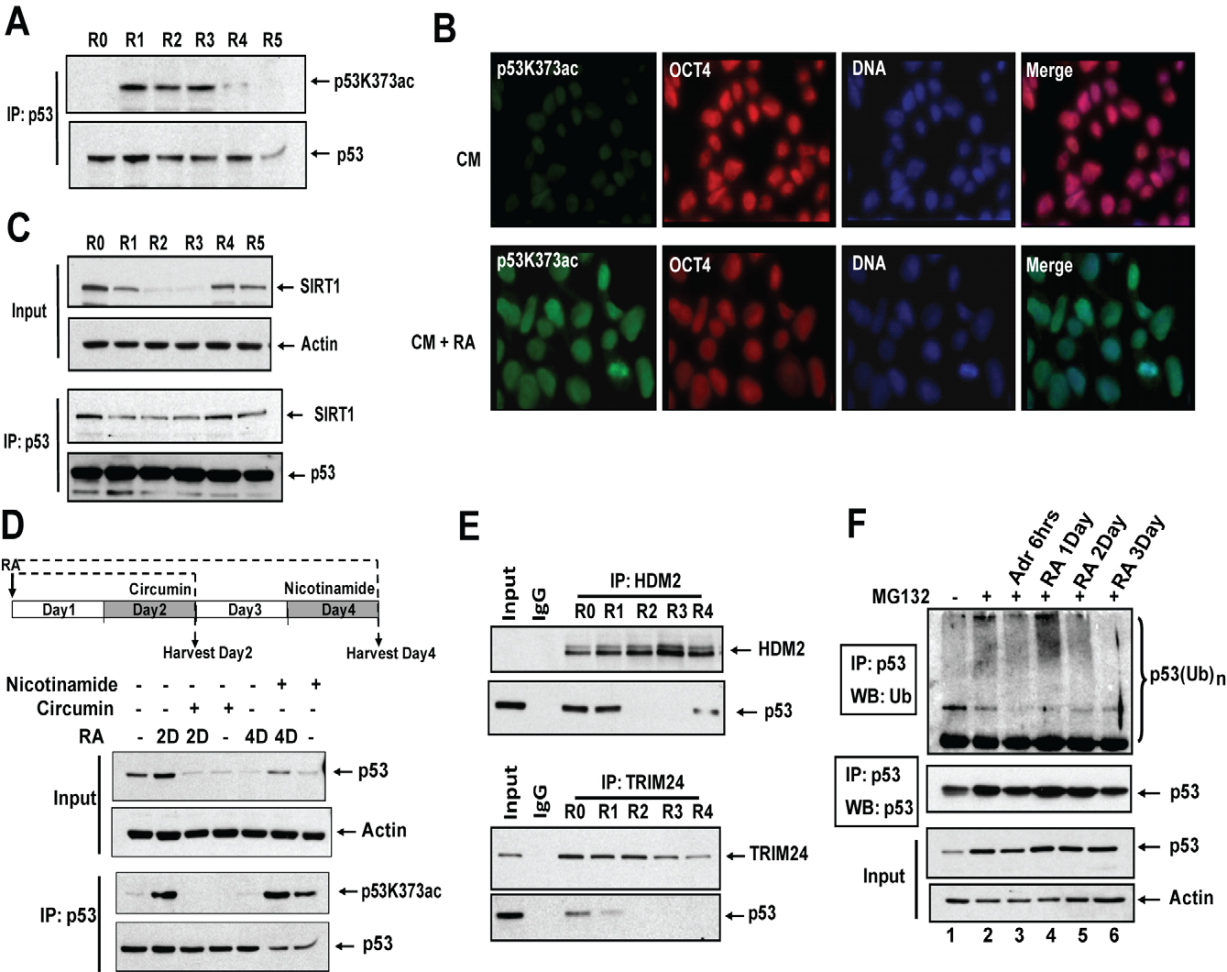
Acetylation of Lys373 leads to stabilization of p53. (A) p53 acetylation. Equal amounts of p53 were immunoprecipitated by adjusting the amounts of total cell lysates prepared from hESCs and probed with p53K373ac antibody. (B) Immunofluorescence. hESCs treated with RA for 3 d were stained with antibodies against p53K373ac and OCT4; nuclei were stained with DAPI. (C) Co-immunoprecipitation. Cell lysates from RA-treated hESCs were immunoprecipitated with p53 followed by Western blotting. (D) p53 acetylation. p53 immunoprecipitated from hESCs cultured under differentiating conditions and treated with either circumin on day 2 or nicotinamide on day 4 and probed with p53K373ac antibody. (E) Co-immunoprecipitation. Cell lysates from differentiating hESCs were immunoprecipitated with HDM2 or TRIM24 antibodies and analyzed by Western blotting. (F) Endogenous p53 ubiquitination. hESCs cultured under differentiating conditions were treated with MG132 + RA or MG132 + Adr; endogenous p53 was immunoprecipitated and probed for ubiquitin (top panel). Same blot was reprobed with p53 antibody to show the equal p53 pull down (bottom panel). (Also see Figure S2.) IP, mmunoprecipitation; Ub, ubiquitin; WB, Western blot.

Stress activation of p53 is primarily attributed to post-translational modification of p53 and increased protein stability [30]. During RA-mediated differentiation of hESCs, p53 gained acetylation at residue lysine 373 (p53K373; Figures 2A, 2B, and S2A), and DNA-damage-associated modifications, such as phosphorylation of p53S15 or p53S46, were not observed (Figure S2A). p53K373 is a known substrate of histone acetyltransferase CBP/p300 [31], and treatment of differentiating hESCs with CBP/p300 inhibitor circumin [32] led to loss of p53K373ac and p53 stability during differentiation (Figure 2D). Increased p53K373ac occurred in parallel with reduced levels of SIRT1, at days 1–3 of RA treatment, suggesting that a pool of p53 escapes deacetylation by SIRT1 (Figure 2C). NAD^+^-dependent histone deacetylase SIRT1 is down-regulated during differentiation of hESCs, as described previously [33]; however, SIRT1 protein levels and p53 interaction recover after differentiation of hESCs (day 4, Figure 2C), as p53 and p53K373ac are restored to low levels (Figures 1 and 2A). Addition of an inhibitor of SIRT1 activity, nicotinamide [34], on day 4 of RA treatment, maintained p53K373ac (Figure 2D). These results suggest that an active acetylation/deacetylation switch regulates p53 during differentiation of hESCs.

Pluripotent hESCs have low p53 levels (Figures 1 and S1C), similar to somatic cells, where p53 levels are regulated by E3-ubiquitin ligases, ubiquitin modification, and proteasomal degradation [35],[36]. HDM2, which is embryonicly lethal when deleted (as *mdm2*) in mice [37], and TRIM24, a negative regulator of p53 identified in mESCs [38], are associated with p53 in pluripotent hESCs but dissociate after RA addition (Figure 2E). Ubiquitin-modified p53 species are detectable at 0–24 h of RA treatment in the presence of inhibitors of the proteasome, MG132 (lanes 2 and 4, Figures 2F and S2B) and lactacystin (Figure S2C). After RA treatment, ubiquitin-modified p53 species decreased in abundance with time of differentiation: compare (lanes 5 and 6, Figures 2F, S2B, and S2C). As a positive control for regulated loss of ubiquitin-modified p53 [39], hESCs were treated in parallel with the DNA-damaging agent adriamycin (Adr) (Figure 2F, lane3, and Figure S2B and S2C). Together, gain of acetylation and loss of ubiquitination transiently increased p53 stability during hESC differentiation.

### The Consequences of p53 Accumulation in hESCs

**Figure 3:**
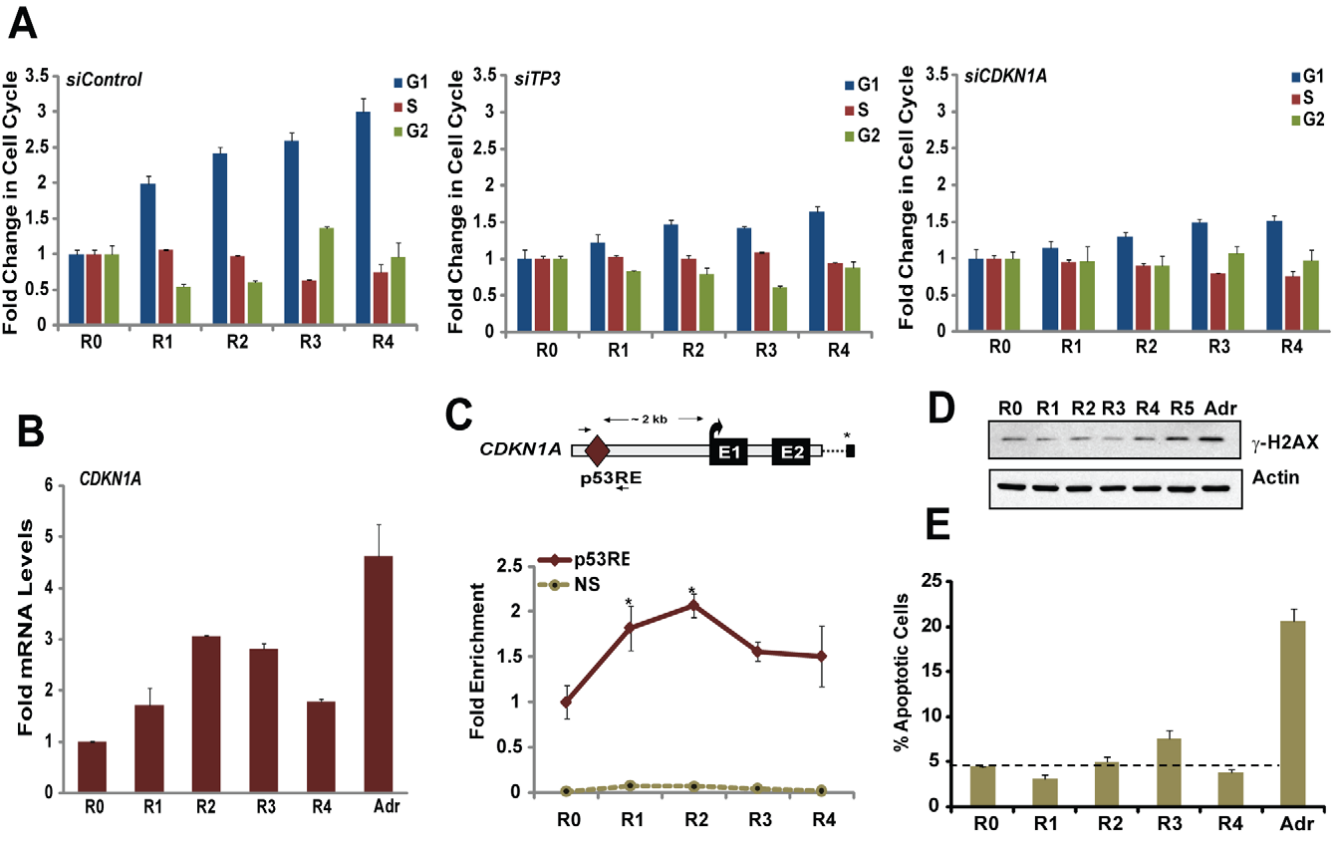
The consequence of p53 accumulation in hESCs. (A) Cell cycle analysis. hESCs transfected with non-target (siControl) or siRNA specific to p53 (*siTP53*) or p21 (*siCDKN1A*) and treated with RA were stained with PI and subjected to flow cytometry analysis to determine DNA content. Results quantitated as fold change in cell cycle are shown. (B) qRT-PCR. RNA from hESCs treated with RA for 4 d or Adr for 6 h were subjected to qRT-PCR assay using primers specific for human *CDKN1A*. (C) ChIP. p53-bound chromatin was immunoprecipitated from hESCs, and p53 enrichment on *CDKN1A* was analyzed by qRT-PCR using primers encompassing p53REs (*, *p*<0.05). Scheme representing location of p53RE and primers used for ChIP-qRT-PCR are shown on the top (asterisk indicates the 3′ end of the gene). (D) hESCs treated with RA or Adr were lysed, and cell lysates were blotted for γ-H2AX. (E) Apoptosis assay. hESCs treated as in (D) were stained with Annexin V and PI, and percent apoptotic cells was determined by flow cytometry (mean ± SEM). (Also see Figures S3 and S4.).

DNA damage of mESCs, where p53 is expressed at high levels and is primarily cytoplasmic, leads to repression of *NANOG* and spontaneous differentiation into other cell types, which undergo p53-dependent apoptosis [28],[40],[41]. However, a previous report shows that, unlike mESCs, exposure of hESCs to DNA damage induces p53-dependent cell cycle arrest rather than differentiation [42]. In order to assess functions of p53 during differentiation of hESCs we performed flow cytometry analysis of the cell cycle in hESCs at time points of exposure to RA, and compared control and p53-depleted hESCs (Figure 3). We efficiently depleted p53, and other targets, with pools of small interfering RNA (siRNA) and a modified siRNA transfection protocol (see Materials Methods for details) that had an average 60% transfection efficiency and that produced an up to 80% reduction in RNA expression (Figure S3).

Flow cytometry showed that 60% of pluripotent hESCs are in S phase, and approximately 10% of hESCs are in G_1_ (time = 0), consistent with a rapid cell cycle (15–16 h) due to truncation of G_1_ [13] (Figures 3A and S4A). During differentiation, hESCs spend increased time in G_1_, slowing down cell cycle over time with RA treatment: at day 4 there is a 3-fold increase in cells in G_1_ (Figure 3A). The accumulation of hESCs in G_1_ continued during differentiation; after 10 d of RA treatment, hESCs attained a cell cycle profile more similar to that of differentiated cells (human foreskin fibroblasts), with more than 60% of cells in G_1_ (Figure S4B). When hESCs were depleted of p53 by siRNA and exposed to RA, the accumulation of cells in G_1_ was attenuated, indicating that p53 plays an integral role in the process (Figures 3A and S4A). The accumulation of hESCs in G_1_ during differentiation stands in contrast to DNA damage, which led to a p53-dependent arrest at the G_2_–M transition and apoptosis with exposure to damage-inducing levels of Adr (Figure S4C–S4E).

**Figure 4:**
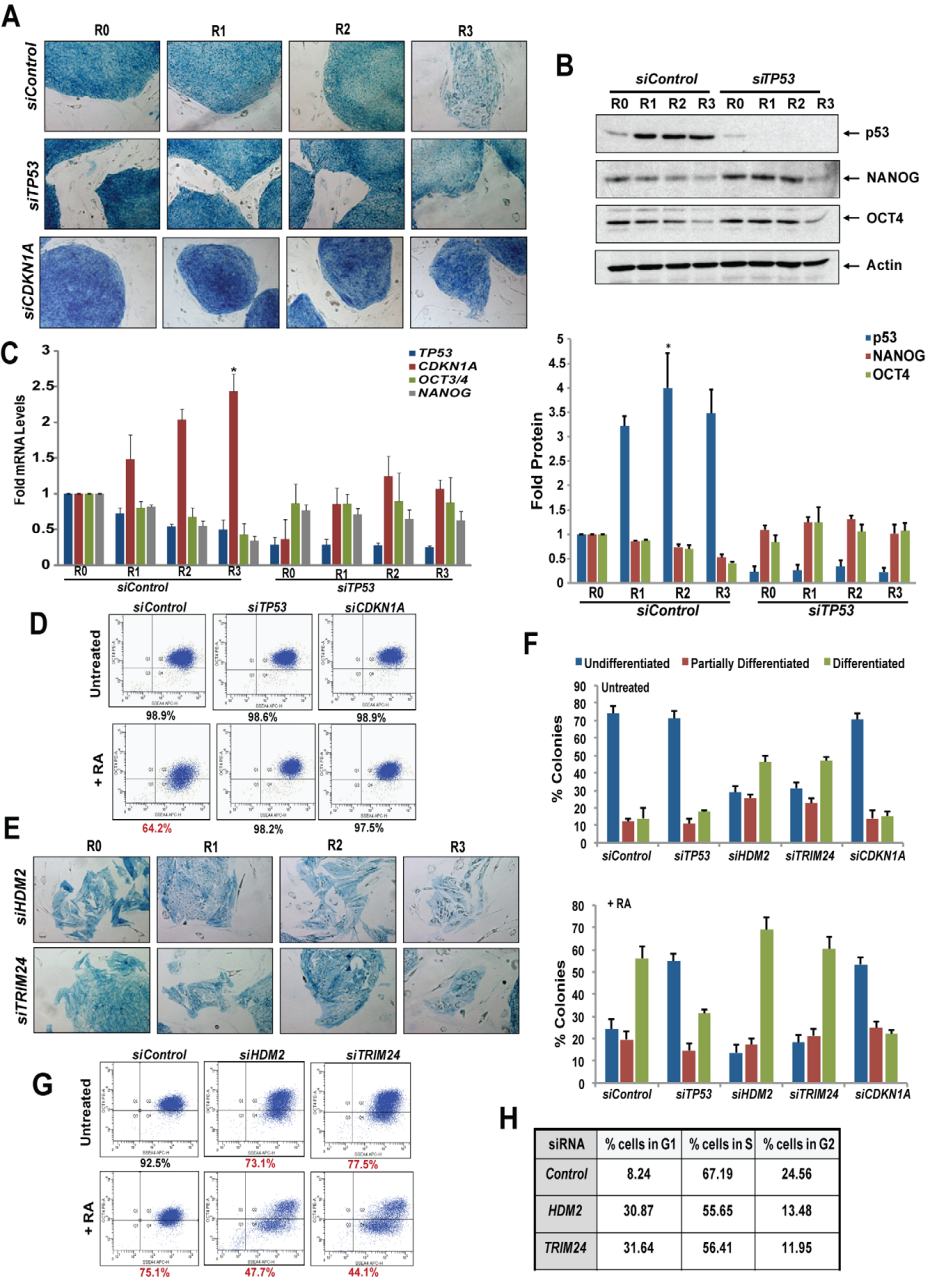
p53 drives differentiation of hESCs. (A) AP staining. hESCs transfected with non-target (siControl) or siRNA specific to p53 (*siTP53*) or p21 (*siCDKN1A*) were treated with RA and stained for AP (blue colonies). (B and C) hESCs transfected and treated as in (A) were used in Western blotting (B) and qRT-PCR (C) analyses. The blots in (B) were quantitated, and average density of three different blots is plotted (bottom panel) (*, *p*<0.05) (mean ± SEM). (D) OCT4 + SSEA4 staining. hESCs transfected with siRNA followed by RA treatment were stained for SSEA4 and OCT4 and subjected to dual flow cytometry analysis. Triplicate samples were analyzed in each experiment, and data analyzed with FACSDiva software. Decreases in fraction of OCT4-positive cells, as compared to siControl untreated, are indicated in red. (E) AP staining. hESCs transfected with *HDM2* or *TRIM24* siRNA were treated with RA and stained for AP. (F) Quantified AP-stained colonies. Date shown are for 50 colonies per treatment in three separate experiments (in [A] and [E]), scored as undifferentiated, partially differentiated, or fully differentiated colonies, mean ± SEM. (G) OCT4 + SSEA4 staining and flow cytometry analysis as in (D) after transfection with siRNA targeting *HDM2* or *TRIM24*. (H) Cell cycle analysis. hESCs transfected with *HDM2* or *TRIM24* siRNA were stained with PI and subjected to cell cycle analysis. (Also see Figures S3 and S5.).

Cell cycle arrest in G1 phase may be mediated by cyclin-dependent kinase inhibitor p21/WAF1, a downstream gene target of p53 [43],[44]. We observed increased expression of *CDKN1A* (p21) (Figure 3B), which was p53-dependent (Figure 4C), in parallel with accumulation of hESCs in G1 phase during differentiation of both WA09 and WA01 hESCs (). p53 directly regulates p21 expression, as chromatin immunoprecipitation (ChIP) analysis revealed RA-induced enrichment of p53 at the distal p53 response element (p53RE) of *CDKN1A* (Figure 3C). The importance of p21 in RA-mediated alteration of the hESC cell cycle was shown by siRNA depletion of *CDKN1A* and loss of hESC accumulation in G_1_ (Figure 3A). An RA-mediated elongation of the G_1_ phase during differentiation of hESCs was marked by a specific increase in unmodified retinoblastoma tumor suppressor protein (non-phosphorylated RB), alongside up-regulated p21 protein (Figure S4F). Previous studies show that RB is hyper-phosphorylated and inactive in self-renewing, cycling hESCs [11],[45].

Interestingly, differentiation-induced p53 did not activate expression of genes *GADD45A* and *BAX*, which are associated with apoptosis (Figure S4G). In contrast, hESCs underwent cell death after exposure to appropriate levels of DNA-damaging agents (Figure 3D and 3E), and showed p53 enrichment on p53REs and increased expression of *CDKN1A*, *MDM2*, *BAX*, and *GADD45A* under these conditions (Figure S4G and S4H). RA-induced p53 and differentiation had little effect on the number of apoptotic hESCs, as shown by Annexin V staining and γ-H2AX levels (Figure 3D and 3E).

### p53 Promotes Differentiation of hESCs

**Figure 5:**
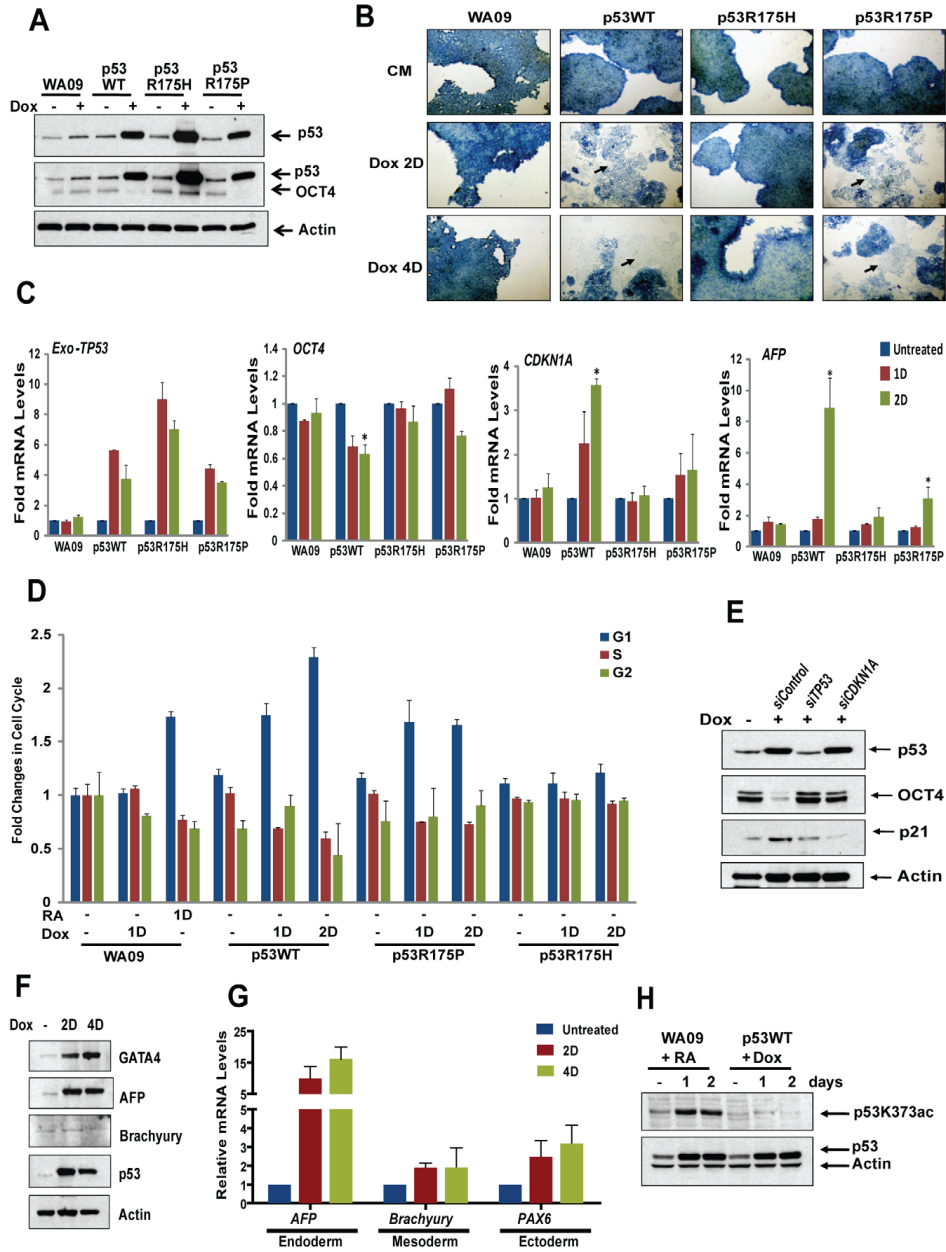
DNA binding activity of p53 is required to induce differentiation of hESCs. (A) hESCs stably expressing p53WT and mutant p53 (p53R175H and p53R175P) under control of tet-inducible promoter cultured in CM + FGF were treated with 100 ng/ml Dox for 2 d. p53 and OCT4 protein levels were analyzed by blotting. (B) AP staining. hESCs in (A) treated with Dox for 2 d (2D) or 4 d (4D) and AP stained. (Arrows indicate differentiated cells.) (C) qRT-PCR. hESCs treated with Dox for 1 d (1D) or 2 d (2D). RNA analyzed by qRT-PCR assay for expression of exogenous *TP53, CDKN1A, OCT4*, and *AFP* (*, *p*<0.05) (mean ± SEM). (D) Cell cycle analysis. hESCs treated with RA for 1 d or Dox for 1 d or 2 d, stained with PI, and subjected to cell cycle analysis (mean ± SEM). (E) hESCs stably expressing p53WT were transfected with siRNA and treated with Dox for 2 d. p53, p21, and OCT4 protein levels were analyzed. (F and G) hESCs expressing p53WT were treated with Dox for 2 d or 4 d, and lysed to analyze protein (F) or RNA (G) for various differentiation markers; AFP and GATA4 (endoderm), Brachyury (mesoderm), and PAX6 (ectoderm). (H) p53 acetylation. Lysates from hESCs treated with RA and Dox-inducible p53WT treated with Dox were blotted for p53K373ac and p53. (Also see Figure S6.).

One approach we used to assess the progression of RA-mediated differentiation was to stain hESC cultures with alkaline phosphatase (AP) at each time point. As previously reported [46], differentiation is marked by loss of AP staining and appearance of cells with a flattened cellular morphology (Figure 4A). Depletion of p53 by siRNA delayed RA-mediated differentiation (Figure 4A and 4B; see also Figure 5E), as more than 60% of hESCs remained undifferentiated after 3 d of RA treatment (AP-stained colonies quantified in Figure 4F). Additionally, with siRNA of p53, pluripotency markers NANOG and OCT4 maintained expression and there was no induction of p21, as compared to cells transfected with non-target siRNA (siControl) (Figure 4B and 4C). These results were confirmed by flow cytometry analysis of hESCs stained with OCT4 and SSEA4, which revealed no reduction in OCT4 staining in cells depleted of p53 compared to only 64% cells positive for OCT4 in siControl hESCs, 3 d after RA treatment (Figure 4D).

Up-regulation of p53 is transient during differentiation of hESCs, as auto-regulation of negative regulators, HDM2 and TRIM24, is triggered by p53 (Figure S5). We transiently transfected pools of siRNA specific for either HDM2 or TRIM24, and achieved ∼70% reduction in levels of gene expression in each case (Figures S3 and S5). Depletion of HDM2 and TRIM24 by siRNA in untreated hESCs increased p53 protein levels (Figure S5A), increased p21 RNA and protein, and decreased OCT4 and NANOG expression (Figure S5B). Depletion of either HDM2 or TRIM24 led to spontaneous differentiation (approximately 50% differentiated colonies) (Figure 4E and 4F), as well as a 4-fold increase in the number of hESCs in G_1_ phase (Figure 4H). Flow cytometry revealed a significant reduction in OCT4-positive cells (Figure 4G), which correlates with siRNA-mediated depletion of HDM2 and TRIM24 (Figure S3). Spontaneous differentiation of hESCs, with increased expression of p53 and p21, occurred even under cell culture conditions where pluripotency is normally maintained and without RA treatment (Figures 4E–4H and S5). This is in sharp contrast to hESCs depleted of p21, which exhibited significantly delayed differentiation (Figure 4A, bottom panel, and Figure 4F), no change in percent of cells positively stained for OCT4 (Figure 4D), and no increase in cells residing in G_1_ (Figure 3A). Expression of pluripotency markers under these conditions further supported the links between p53, p21, and differentiation (Figure S5B).

### DNA Binding Is Required for p53-Mediated Regulation of Differentiation

We established a system in hESCs where expression of p53 is controlled in a dose-dependent manner without addition of RA. Lentiviral constructs, which express wild-type or mutant p53 with an IRES-GFP reporter under control of a tetracycline (doxycycline [Dox])–responsive promoter, were introduced into hESCs and selected for stable integration. Cell lines with regulated expression of wild-type p53 (p53WT) or p53 mutated within its DNA-binding domain (p53R175H and p53R175P) were positive for both OCT4 and SSEA4 stem cell markers, as assessed by flow cytometry analysis, in the absence of Dox (Figure S6A) and exhibited Dox-regulated expression of p53 (Figure 5). In the absence of RA, Dox-induced expression of p53WT led to loss of OCT4 expression. In contrast, expression of a mutated form of p53, incapable of binding to DNA and regulating p53 target gene expression (p53175H), did not correlate with decreased OCT4 even though p53R175H protein levels are higher than those of p53WT (Figure 5A). Interestingly, Dox induction of a mutated form of p53, p53R175P, known to induce cell cycle arrest but not apoptosis in mouse models [47],[48] led to loss of OCT4 expression, although the decrease was smaller than the significant reduction induced by p53WT (Figure 5A and 5C). The dichotomy between responses of hESCs exposed to p53R175H versus p53R175P supports functions of p53 in the activation of cell cycle arrest, without apoptosis, during differentiation of human cells.

Further analysis of differentiation driven by conditionally regulated p53, in the absence of RA, showed that p53 target genes, *CDKN1A* and *HDM2*, were activated over a time course of Dox exposure (Figures 5C and S6B). In parallel, pluripotency marker gene expression of *KLF4, NANOG*, and *OCT4* was significantly reduced with length of exposure to Dox (Figures 5C and S6C). Dox treatment led to a ∼4- to 5-fold induction in exogenous wild-type and mutant p53 RNA, with an insignificant change in endogenous *TP53* levels (Figures 5C and S6B). Differentiation occurred as marked by a gain in *AFP* expression and flattened cell morphology, with loss of AP staining in hESCs expressing p53WT or p53R175P but not p53R175H (Figure 5B and 5C). Ectopic expression of p53WT and p53R175P led to an increase of cells in G_1_, similar to induction by RA treatment; however, p53R175H did not affect the cell cycle profiles of hESCs (Figure 5D). Induction of p53WT in hESCs led to expression of endoderm markers GATA4 and AFP, as well as ectoderm marker PAX6 (Figure 5F and 5G). However, these cells did not express mesodermal marker Brachyury (Figure 5F and 5G). Differentiation is specific to p53, as depletion of Dox-induced p53 by siRNA, added at *t = *0, rescues OCT4 protein expression (Figure 5E). Similarly, after siRNA-mediated depletion of p21 in hESCs over-expressing p53, OCT4 protein levels were rescued, further confirming p21 as a mediator of p53-induced differentiation of hESCs, whether induced by exogenous p53WT or by RA treatment (Figure 5E).

Interestingly, Dox-induced p53 is not acetylated at K373, as is readily detectable when RA is used to induce equivalent levels of endogenous p53 and differentiation of hESCs (Figures 2 and 5H). This finding suggests that ectopic induction of p53 circumvents K373 acetylation, which promotes release of endogenous p53 from negative regulatory proteins during RA-induced differentiation (Figure 2). Taken together, these results support a view of p53 as a critical regulator of hESC differentiation, capable of acting in the absence of RA and other stimuli that may be induced by RA treatment. The role of cell cycle regulators in this process is underscored by expression of mutated forms of p53 that specifically regulate genes that lead to G_1_ arrest but are unable to regulate apoptosis in mouse models [47].

### p53 Regulates *miR-34a* and *miR-145* to Drive Differentiation of hESCs

To understand the mechanism underlying p53-mediated differentiation of hESCs, we performed high-throughput ChIP sequencing analysis of hESCs incubated with RA (unpublished data). Putative p53 targets included miRNAs; among these, we focused on *miR-34a* and *miR-145* as likely significant in the p53-mediated regulation of hESCs. In somatic cells, *miR-34a* acts in a feed-forward loop of p53 control. In response to stress stimuli, p53 is activated and induces expression of *miR-34a*, which in turn represses negative p53 regulator SIRT1 to augment p53 activation [49],[50], and CyclinD1 and CDK6 to support cell cycle arrest [51]. SIRT1 deacetylates p53, which decreases the ability of p53 to bind DNA and regulate gene expression [52], as we also show in pluripotent hESCs (Figure 2). Regulation of *miR-34a* and its downstream targets in ESCs has not been previously reported, to our knowledge. In contrast, a role for *miR-145* in differentiation of hESCs is known, where *miR-145* acts by negatively regulating levels of pluripotency genes, *OCT4, SOX2*, and *KLF4* [53]. *miR-145* is known to be a p53 target in somatic cells [54]; however, the mechanisms that lead to *miR-145* up-regulation during differentiation of hESCs have not been defined.

**Figure 6:**
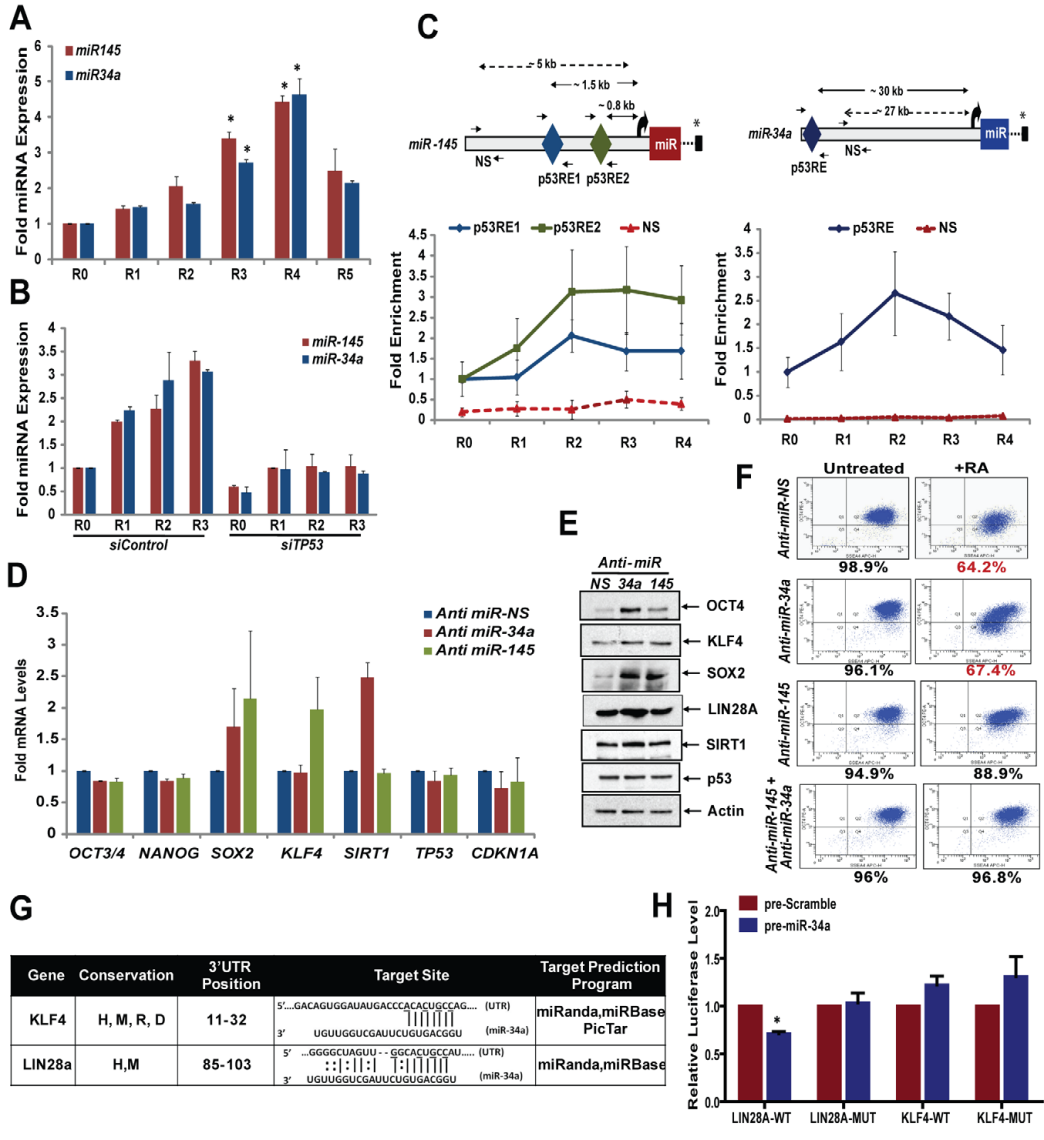
p53 regulates *miR-34a* and *miR-145* to drive differentiation of hESCs. (A and B) miRNA-TaqMan assay. miRNAs were analyzed using total RNA from hESCs with probes specific for human *miR-34a* and *miR-145* and were normalized to RNU6B as internal control (*, *p*<0.01). (B) Total RNA prepared from hESCs transfected with siRNA was analyzed for expression of *miR-34a* and *miR-145*. Data are presented as mean ± SEM. (C) ChIP. p53-bound chromatin was immunoprecipitated from hESCs, and p53 enrichment on *miR-34a* and *miR-145* promoters was analyzed by qRT-PCR (*, *p*<0.05). Scheme representing location of p53RE and primers used for ChIP-qRT-PCR are shown on the top (asterisk indicates the 3′ end of the gene). Data are presented as mean ± SEM. (D and E) Anti-miRNA assay. hESCs transfected with anti-miRNA oligonucleotides for non-specific (NS), *miR-34a*, and *miR-145* were subjected to qRT-PCR assay (D) and Western blotting (E) (mean ± SEM). (F) OCT4 + SSEA4 staining. hESCs transfected as in (D) followed by RA treatment for 3 d were stained for SSEA4 and OCT4 and subjected to flow cytometry analysis. Decreases in fraction of OCT4-positive cells, as compared to untreated control, are indicated in red. (G) Summary of *miR-34a* target sites in the 3′ UTRs of *KLF4* and *LIN28A*. D, dog; H, human; M, mouse; R, rat. The underlined nucleotides in miRNA target sites were mutated in the mutant 3′ UTR constructs. (H) Luciferase assay. HEK293 cells were transfected with luciferase plasmids containing wild-type (WT) or mutated (Mut) 3′ UTRs along with miRNA precursors for scrambled or *miR-34a*. Relative luciferase levels were calculated, and Student's *t* test was used to compare the datasets (*, *p*<0.01). Error bars represent standard deviation for three independent experiments. (Also see Figure S7.).

In response to RA treatment and differentiation of hESCs, *miR-34a* and *miR-145* were significantly up-regulated in a p53-dependent manner (Figure 6A and 6B), an induction which also occurs with a DNA-damaging agent, Adr (Figure S7A). RA treatment led to a time-dependent enrichment of p53 at predicted p53REs of both *miR-34a* and *miR-145* (Figure 6C), in parallel with the transient activation of p53. Interestingly, p53 enrichment on two identified p53REs of *miR-145* exhibited distinct patterns during differentiation compared to DNA damage: p53 accumulation occurred at both p53REs but was stronger on the proximal p53RE (p53RE2) during differentiation and on the distal p53RE (p53RE1) after DNA damage (Figures 6C and S7B). Introduction of small inhibitory oligonucleotides to counter expression of targeted miRNAs (anti-miRNAs), anti-*miR-34a* and anti-*miR-145*, resulted in ∼80% miRNA depletion (Figure S6C), and had specific effects on expression of stem cell factors: inhibition of *miR-34a* led to increased expression of OCT4, KLF4, LIN28A, and SOX2 proteins, and to a lesser extent SIRT1 (Figure 6E), as well as *SOX2* and *SIRT1* RNA (Figure 6D). Inhibition of *miR-145* induced protein levels of OCT4, SOX2, and KLF4, as well as increased RNA expression of *SOX2* and *KLF4* (Figure 6D and 6E). Quantitative determination of OCT4/SSEA4-positive cells by flow cytometry analysis revealed that hESCs could differentiate with RA after inhibition of *miR-34a* but not in the presence of anti-*miR-145* (Figure 6F). Depletion of both miRNAs significantly delayed differentiation of hESCs, as ∼97% of hESCs remained OCT4-positive 3 d after RA treatment (Figure 6F), indicating the significance of these miRNAs during differentiation.

Depletion of *miR-145* also significantly affected accumulation of hESCs in G_1_ after RA treatment (Figure S7D). *miR-145* targets c-Myc [54], which is known to repress p21 [55]; thus, *miR-145* represses pluripotency factors and likely contributes to regulation of the hESC cell cycle by decreasing *c-Myc* and indirectly activating p21 during differentiation. In silico analysis by TargetScan [56], PicTar [57], miRanda [58], and miRBase [59] of genes potentially regulated by *miR-34a* and *miR-145* identified several genes significant to ESC biology (Figure S7E). Pluripotency genes targeted by *mir-145* are known [53]; additionally, we found that *mir-34a* has predicted target sites within the 3′ UTRs of *KLF4* and *LIN28A*, which are conserved across species (Figure 6G). To investigate whether *KLF4* and *LIN28A* are directly targeted by *miR-34a*, we engineered luciferase reporters that have either the wild-type 3′ UTRs of these genes, or mutated 3′ UTRs with a 4-bp mutation in the predicted target sites. The luciferase reporters were cotransfected with miRNA precursor (pre-miRNAs) mimics, which are processed into mature miRNAs in HEK293 cells. A scrambled precursor with no homology to the human genome was used as a control. The pre-miRNA mimic of *miR-34a* (pre-*miR-34a*) significantly reduced the luciferase activity of the wild-type *LIN28A* reporter (∼30%), compared to the scrambled precursor control (two-tailed Student's *t* test; Figure 6H), and did not alter activity of mutated reporters (Figure 6H). Repression was specific to *LIN28A*, as there was no significant effect of pre-*miR-34a* transfection on the *KLF4* reporter. These results suggest that *miR-34a* directly targets sites within the *LIN28A* 3′ UTR. Taken together, p53-activated miRNAs decrease expression of major stem cell factors to oppose self-renewal of hESCs, as well as inhibiting *SIRT1*, a negative regulator of p53. Thus, p53-mediated regulation of miRNAs reinforces and expands the direct effects of p53 in regulation of the cell cycle during differentiation of hESCs.

## Discussion

*TP53* is mutated in more than half of all human cancers, and maintains genomic stability in somatic cells, primarily as a stress-responsive transcription regulator of genes that control cell cycle arrest and apoptosis [60],[61]. Functions of p53 in cellular metabolism, homeostasis, and development are less understood, but are increasingly appreciated [62],[63]. In adult stem cells, p53 negatively regulates proliferation and self-renewal of neural stem cells and hematopoietic stem cells to maintain their quiescent state [64],[65]. A role for p53 in ESC modulation was first suggested by a report that p53 directly represses *Nanog* in mESCs [40]. Likewise, p53 functions in apoptosis and differentiation of hESCs were previously reported, but no clear mechanisms were revealed [66],[67].

**Figure 7:**
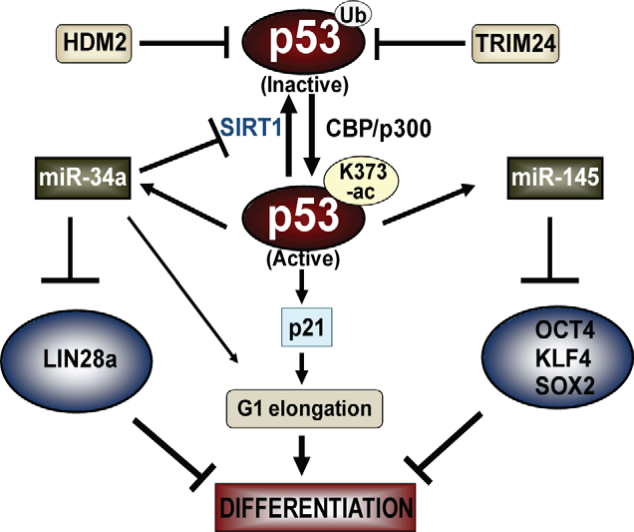
Model depicting role of p53 in inducing differentiation of hESCs. In pluripotent hESCs, p53 is negatively regulated by HDM2 and TRIM24. Differentiation induces acetylation at Lys373 of p53 via CBP/p300, p53K373ac then activates transcription by binding to p53REs on *CDKN1A* (p21), *miR-34a*, and *miR-145*. Induction of p21 leads to p53-dependent elongation of G_1_ phase, whereas induction of *miR-34a* supports G1 elongation, blocks deactivation of p53 by targeting the deacetylase SIRT1, and counteracts pluripotency by targeting *LIN28A*. On the other hand, *miR-145* targets OCT4, KLF4, and SOX2 and antagonize pluripotency. Thus, p53 exerts a cumulative pro-differentiation effect by elongating hESC G_1_ phase via p21 and synergistically up-regulating *miR-34a* and *miR-145* to counteract pluripotency. Ub, ubiquitin.

Here, we show that p53 actively promotes differentiation of hESCs and does so by mechanisms distinct from direct regulation of *NANOG* transcription (Figure 7). In contrast to in mESCs, human p53 is localized in the nucleus of hESCs at a low concentration and in a deacetylated state. In response to differentiation signals, SIRT1 is down-regulated [33], allowing p53 to be acetylated at Lys373, a target of CBP/p300. Acetylation of p53 activates its functions as a transcription factor [68], and relieves p53 from HDM2- and *TRIM24* (shown here)– mediated ubiquitination and degradation [34]. The importance of p53 concentration and its regulation was shown by the significant levels of differentiation that occur either in response to siRNA-mediated depletion of *MDM2* and TRIM24 or by ectopic expression of p53. In these cases, differentiation occurs in the absence of RA and in medium that normally maintains stem cells as such.

Comparison of cell cycle profiles during RA-mediated differentiation and DNA damage conditions highlights the diverse roles played by p53 to restrict cell division and initiate either differentiation or DNA repair, respectively. Differentiation-activated p53 binds to the p53REs of downstream gene target *CDKN1A* to promote a G_1_ block that effectively elongates G_1_ and lengthens the cell cycle of hESCs, with minimal induction of apoptosis. The pattern of post-translational modifications of p53 and the accumulation of hESCs in G_1_ induced by RA are in contrast to arrest of Adr-treated hESCs at G_2_–M of cell cycle in a classical, p53-mediated DNA damage response [60]. We find that p53, transiently activated during differentiation, regulates cell cycle but does not induce significant apoptosis. Although selectivity of p53 in activating arrest of cell cycle versus apoptosis remains incompletely understood [30], our findings for hESC differentiation recapitulate the specificity previously shown in mouse models that express specific point mutants of p53 [48],[69] and suggest that these mechanisms are highly conserved. Differentiation of hESCs is significantly delayed when *TP53* and/or *CDKN1A* levels are reduced, as shown by AP staining, cell cycle analysis, and expression of markers of pluripotency in hESCs transfected with siRNAs. Recently, Dox-inducible exogenous expression of p21 in hESCs was shown to induce cell cycle arrest and massive hESC differentiation [70], further supporting that induced levels of p21 and control of cell cycle are required for hESCs to differentiate.

A number of p53 downstream target genes have been extensively studied, especially in transformed cells, and non-coding RNAs regulated by p53 are now being identified [71]. Roles of miRNAs during hESC differentiation or reprogramming of somatic cells have recently been reported [24],[53],[72]–[76], with specific signatures of miRNAs shown in distinct stem cell states of pluripotency regulated by p53 [77]. A known p53 target, *miR-34a*, was shown to increase p53 activation by repressing SIRT1 in cultured cells [49] but was not previously linked to hESCs. *miR-145* was discovered to be a direct repressor of pluripotency factors, but was not shown to be regulated by p53 in stem cells [53]. Here we show that p53 activates *miR-34a* and *miR-145* expression during RA-mediated differentiation of hESCs. Expression of these miRNAs, directly induced by chromatin interaction of p53 at p53REs, impacts a network of target transcripts that control pluripotency, either directly or indirectly. Further, during preparation of this manuscript, Choi et al. [78] showed that *miR-34a* provides a barrier to somatic cell reprogramming. This study offers further support for our finding that *miR-34a* antagonizes pluripotency of hESCs and has pro-differentiation functions in stem cell biology. We found that *miR-145* has a more significant role in differentiation of hESCs, with *miR-34a* acting to augment its functions. However, since DNA damage induced miRNAs *miR-34a* and *miR-145* but did not promote accumulation of hESCs in G_1_ and differentiation of hESCs, as seen with RA treatment or ectopic p53 expression, it is clear the miRNAs alone are insufficient to induce differentiation of hESCs. We showed that p53 activation is transient during differentiation of hESCs; thus, activation of miRNAs that repress stem cell factor expression broadens the impact of p53 activation and may prevent “backsliding” to pluripotency, once p53 returns to its normally low concentration during differentiation to a committed state.

Recently, the creation of *TP53^−/−^* hESCs by homologous recombination showed that loss of p53 promotes pluripotency, a role for p53 conserved in both murine and human ESCs [79]. However, *TP53^−/−^* hESCs contribute to all three germ layers during teratoma formation in SCID mice, perhaps because of compensation by structurally similar members of the p53 family, p63 and p73. Deletion of all p63 and p73 isoforms in mice reveals critical roles in development and differentiation [80],[81]. p63 and p73 can bind to canonical p53 DNA-binding sites and regulate transcription from p53-responsive promoters, in the presence or absence of p53 itself [82]–[84]. Compensation may be incomplete, as p53-null mice exhibit some developmental anomalies, such as a high percentage of exencephaly in females, and specific genes exhibit altered p53-regulated gene expression during development [85]–[87].

The establishment of elongated Gap-phase timing in stem cells, more similar to that in somatic cells, was previously proposed as a requirement for reception of differentiation signaling [11]. Our studies show that p53 is integral in this process and actively promotes differentiation of hESCs, in the absence of cellular stress or DNA damage. The collective effects of p53 activation elongate the G_1_ phase and antagonize pluripotency by induction of *miR-34a* and *miR-145* (Figure 7). Importantly, activation of p53 during differentiation of hESCs is transient, allowing later stages of growth and differentiation, while p53-induced miRNAs regulate a network of genes that bolster forward progression to differentiate. How these findings in ESCs may be relevant in adult and tumor stem cells—where they may be channeled toward therapeutic applications to restructure cell cycle and regulate a network of miRNAs—is an important area for further study.

## Materials and Methods

### Cell Culture

hESCs (WA09 and WA01) were obtained from National Stem Cell Bank and cultured according to the protocol from WiCell Research Institute. Briefly, WA09 cells were maintained in hESC culture medium on γ-irradiated mouse embryonic fibroblasts (MEFs) prepared using WiCell instructions. hESCs ranging from passage number 32–38 were used for all of our experiments. hESC complete culture medium is composed of DMEM/F12 supplemented with 20% knockout serum replacement, 1 mM L-glutamine, 1% nonessential amino acids, 4 ng/ml human FGF2 (all from Invitrogen), and 0.1 mM 2-mercaptoethanol (Sigma). The medium was changed daily, and cells were passaged every 4–6 d with 1 mg/ml Collagen IV (Invitrogen). For differentiation studies hESCs were cultured in differentiation medium (hESC medium without FGF) containing 1 µM RA for 5 d, with fresh medium change daily. hESCs were also maintained as feeder-free cultures on hESC qualified Matrigel (BD Biosciences) in mTeSR1 medium (Stemcell Technologies) and MEF conditioned medium (CM). Passage 32 hESCs were grown on mTeSR1 medium for five passages. hESCs were cultured on Matrigel following manufacturer's instructions and received fresh mTeSR1 medium daily, and cells were passaged every 4–6 d with 1 mg/ml Dispase (Stemcell Technologies). For differentiating hESCs cultured on mTeSR1, 1 µM RA was added to homemade MEF CM (without additional FGF). CM was prepared in our facility by culturing γ-irradiated MEFs in complete hESC culture medium for 24 h, collected daily, filtered, and freezed at −20°C. FGF was added to CM before use to a final concentration of 10 ng/ml to culture the cells grown on Matrigel under pluripotent conditions.

### RNA and miRNA Knockdown

hESC colonies were grown on Matrigel in six-well plates. siRNA targeting human *TP53*, *CDKN1A* (p21), *HDM2*, *TRIM24* (Table S1) and non-target (control) were purchased from Dharmacon and anti-miRNA oligonucleotides targeting human *miR-34a*, *miR-145*, and *miR-nonspecific* were purchased from Applied Biosystems. 75 nM siRNA or 75 nM anti-miRNA oligonucleotides were transfected twice into cells using Lipofectamine2000 (Invitrogen) transfection reagent according to manufacturer's protocol within a period of 5 d. The first siRNA transfection was performed 24 h after splitting the cells, followed by medium change 6 h post-transfection. 36 h after the first siRNA transfection, cells were cultured in differentiation medium (−FGF, +RA) for 3 d and harvested to analyze protein, RNA, AP, or cell cycle status. Cells were transfected one more time with siRNA on the beginning of day 2 of RA treatment to maintain the knockdown efficiency. To determine the transfection efficiency these siRNAs were cotransfected with siGLO Green (FAM), also purchased from Dharmacon. Twenty-four hours post-transfection cells were either visualized by microscopy or subjected to flow cytometry analysis to determine the percent of cells transfected. See Figure S3 for percent transfection and knockdown efficiencies of siRNAs. In case of anti-miRNA transfections, cells were harvested 24 h post-transfection for RNA and miRNA analysis and 48 h post-transfection for analyzing protein levels.

### Generating hESCs Integrated with tet-Inducible *TP53*

WA09 cells were cultured in mTeSR1 medium as described above. At each passage (Accumax, Millipore) cells were treated for 24 h with 1 µM Y-27632 (Rock inhibitor; Alexis). Lentiviral supernatants were produced by transfecting 293T cells with the pSAM2.gw vector, pCMV-VSV-G and pCMV-ΔR8.91, using Fugene HD transfection reagent (Roche) and collected 48 h post-transfection. WA09 rtTA cells were generated by spin infection of WA09 cells with Lenti-rtTA [88] at 2,500 rpm at 37°C for 90 min with polybrene (4 µg/ml), and incubated at 37°C for an additional 2 h prior to medium replacement with mTeSR1. The WA09 rtTA cells were subsequently transduced with listed pSAM2.gw plasmid as described above. Enrichment of infected cells was performed by FACS sorting (FACSAria II, BD Biosciences) for GFP-positive cells (to a final enrichment of about 80%), following low dose transient induction (addition of 0.05 µg/ml Dox [Sigma]) for 8 h. About 16 h before the sort, the Dox was removed, and the cells were incubated with regular mTeSR1 medium. Positive cells were sorted, plated in fresh mTeSR1, and expanded for secondary/tertiary enrichment or testing.

### Plasmid Generation

pSam2.gw was generated by replacing the ubiquitin C promoter from FUGW [89] with a second generation tetracycline response element [90] , inserting a Gateway positive/negative selection cassette flanked by attR1 and attR2 sequences (Invitrogen), and replacing GFP with the IRES-GFP sequence from pMSCV-IRES-GFP [91]. All constructs to be expressed were then cloned into pENTR1a (Invitrogen) and transferred into pSAM2.gw by Gateway recombination.

### RNA Isolation and Real-Time PCR Analysis

RNA was isolated using TRIzol Reagent (Invitrogen) and miRVana Isolation Kit (Ambion) as per manufacturer's specifications. For RNA analysis 500 ng of total RNA was treated with DNase I, and cDNA was synthesized as previously described [38] and amplified with human-gene-specific primers (Table S2) with Power SYBR Green PCR Master Mix (Applied Biosystems). Primers targeting the 3′ UTR were used to detect endogenous *TP53*, and primers targeted to coding region were used to detect Dox-induced exogenous *TP53*. The average threshold (Ct) was determined for each gene and normalized to *Actin* mRNA level as an internal normalization control. The miRNA levels were assayed with the TaqMan gene expression assays and TaqMan Universal PCR Master Mix in an Applied Biosystems PRISM 7900HT Fast Real-Time PCR System according to the manufacturer's instructions. Briefly, reverse transcription of 10 ng of total RNA by the High-Capacity cDNA Archive Kit (Applied Biosystems) was followed by 18 cycles of pre-PCR and 40 cycles of real-time PCR using TaqMan Fast Universal PCR Master Mix. miRNA levels were normalized against the control RNU6 probe.

### Western Blotting

hESCs were lysed in RIPA buffer (50 mM Tris [pH 8.0], 150 mM NaCl, 1% NP-40, 0.5% deoxycholic acid, 0.1% SDS, 1 mM PMSF, and 1 mM Na-Vanadate) supplemented with protease inhibitor cocktail (Calbiochem) and phosphatase inhibitor cocktails I and II (Sigma). The protein concentration was estimated by the Bradford Protein Assay Kit (Bio-Rad). 50 µg of cell lysate was then separated via sodium dodecyl sulfate polyacrylamide gel electrophoresis (SDS-PAGE) and transferred to nitrocellulose membrane. The membrane was blocked with 5% milk, and protein levels were analyzed by immunoblotting with anti-p53 (DO1), anti-OCT4, anti-SIRT1, anti-SIN3A (Santa Cruz Biotechnology), anti-NANOG, anti-KLF4 (R&D Systems), anti-SOX2, anti-p53K373ac, anti-p53S15ph, anti-p53S46ph, anti-p53K320ac (Cell Signaling Technologies), anti-p21 (BD Pharmingen), anti-pRB (Oncogene Sciences), anti-HDM2 (Calbiochem), anti-TRIM24 (Novus Biologicals), anti-γ-H2AX (Trevigen), and anti-Actin (GeneTex Biotechnology), followed by corresponding HRP-tagged secondary antibody (GE Healthcare).

### Co-Immunoprecipitation Analysis

hESCs grown on inactivated MEF feeders were cultured in complete hESC medium or treated with RA for 5 d in differentiation medium, and the cells were harvested and lysed in RIPA buffer. 0.5 mg of cell lysates was used for immunoprecipitation with primary antibody. The extract was incubated with 2.5 µg of antibody overnight at 4°C with shaking. Forty microliters of washed Protein A bead suspension (GE Healthcare) was added, and the extract was incubated for additional 1 h at 4°C with shaking. Immune complexes were washed twice with RIPA buffer, boiled with 1× SDS sample dye, and resolved on 10% SDS-PAGE gel followed by immunoblotting with corresponding antibodies.

### Subcellular Fractionation

hESCs were grown on 100-mm tissue culture plates in either complete hESC medium or in differentiation medium (−FGF, +RA) for 4 d. At the end of treatment, cells were washed twice with ice-cold PBS, scraped in PBS, and centrifuged at 500 rpm for 5 min. Biochemical fractionation of cells was done using the Nuclear Extract Kit (Active Motif) following the manufacturer's protocol. Briefly, the cell pellet was resuspended in 1× hypotonic buffer (cytoplasmic buffer) supplemented with complete protease inhibitor mixture (Calbiochem), incubated for 15 min at 4°C, vortexed in the presence of detergents, and centrifuged briefly. The supernatant (cytoplasmic fraction) was collected into a prechilled microcentrifuge tube. The nuclear pellet was washed twice with the cytoplasmic buffer, followed by resuspending in the lysis buffer supplemented with 1 mm dithiothreitol and protease inhibitors. The suspension was incubated on a rocking platform at 4°C for 30 min, vortexed briefly and centrifuged for 10 min at 14,000 *g* at 4°C, and the supernatant (nuclear fraction) was collected. The protein concentration was determined using the protein assay reagent (Bio-Rad). 50 µg of the cell fractions was resolved on a 10% SDS-PAGE gel, Western blotted, and probed with anti-p53, anti-OCT4, and anti-NANOG antibodies. To confirm the purity of subcellular fractionation, the extracts were immunoblotted with cytoplasm-specific anti-LDH antibody (Chemicon) and nucleus-specific anti-TBP antibody (Santa Cruz Biotechnology).

### Immunostaining

hESC colonies were grown on Matrigel-coated coverslips in mTeSR1 or MEF complete CM (CM + FGF, 10 ng/ml) or CM differentiation medium (+RA, 1 µM) for 3 d. Cells were fixed with 4% paraformaldehyde in water for 15 min at room temperature (RT). Cells were washed three times with PBS and blocked with blocking buffer (10% normal goat serum in PBS) for 2 h at RT, incubated with anti-p53 and anti-OCT4 antibodies (Santa Cruz Biotechnology) overnight at RT, and washed twice in PBST (PBS + Tween20). The cells were then incubated with the secondary antibody (Alexa-Fluor goat anti-rabbit 488 for OCT4 and Alexa-Fluor goat anti-mouse 546 for p53; Molecular Probes) for 45 min in the dark at RT, followed by two washes with PBST. Coverslips with cells were then mounted on glass slides using Antifade mounting reagent from the Slowfade Antifade Kit (Molecular Probes). The cells were examined and photomicrographed using an Olympus confocal microscope.

### ChIP

hESCs grown on inactivated MEF feeders were cultured in complete hESC medium or treated with RA upto 4 d in differentiation medium and were collected, PBS washed, and crosslinked in 1% formaldehyde for 10 min at RT. After glycine followed by PBS wash, cells were lysed using lysis buffer (5 mM HEPES [pH 8.0], 85 mM KCl, 0.5% NP40) supplemented with protease inhibitor (Calbiochem). After removal of cytoplasmic extract, remaining cell pellet was lysed in nuclear lysis buffer (50 mM Tris-HCl [pH 7.5], 10 mM EDTA, 1% SDS) and protease inhibitor. Lysates were sonicated with glass beads (Sigma) ten times for 10-s pulse on ice to obtain DNA fragments of average length under 500 bp. After centrifugation, the supernatant was preabsorbed with 40 µl of Protein A beads (GE Healthcare) and IgG for 2 h, then incubated with 5 µg of p53 antibody (Santa Cruz Biotechnology) or control IgG (Millipore) overnight at 4°C. The immunocomplex was collected on Protein A beads and washed, and bound DNA was eluted and reverse crosslinked overnight at 65°C. The DNA region of interest was detected by SYBR quantitative real-time PCR (qRT-PCR) using primers encompassing p53REs on the respective gene (see Table S3 for sequence of primers used for ChIP-qRT-PCR).

### p53 Acetylation Analysis

hESCs grown on inactivated MEF feeders were cultured in complete hESC medium or treated with RA for 5 d in differentiation medium, and the cells were harvested and lysed in RIPA buffer. Equal amounts of p53 were pulled down by varying the amounts of total lysates from each treatment and probed with p53K373ac antibody. In order to analyze the effects of CBP/p300 on RA-mediated acetylation of p53, hESCs were treated with RA for 2 d, and 25 µM circumin (Sigma) was added 24 h before harvesting on day 2 [32]. Similarly, to inhibit the deacetylase activity of SIRT1 on p53, 5 mM nicotinamide (Sigma) was added 24 h before harvesting the cells treated with RA for 4 d [34].

### p53 Ubiquitination Assay

hESCs were treated with proteasome inhibitor, MG132 (Calbiochem), or lactacystin (Calbiochem) for 6 h prior to lysis in RIPA buffer supplemented with 10 mM iodoacetamide (GE Healthcare) and protease inhibitors. Endogenous p53 was immunoprecipitated from 1 mg of protein lysate using p53 (DO1) antibody and immunoblotted with anti-ubiquitin antibody (Sigma). The blot was reprobed with p53 to confirm the equal p53 pull down.

### Alkaline Phosphatase Staining

hESC colonies were fixed in 2% formaldehyde (Sigma) and stained with Vector Blue Alkaline Phosphatase Substrate Kit I (Vector Labs) following manufacturer's instructions. The self-renewing colony stains positive for AP, but differentiated colonies stain less or negative for AP.

### Cell Cycle Analysis and Apoptosis Assay

For cell cycle analysis, 1−5×10^5^ cells from each sample were trypsinized to make single cell suspension, washed with PBS, and fixed in 95% ethanol (Sigma). The cells were then treated with RNaseA and stained with propidium iodide (PI) (Calbiochem) as per manufacturer's instructions. 20,000 events were analyzed on an Epics XL flow cytometer (Coulter), and cell cycle distribution was analyzed by the ModFit LT program. For apoptosis assay, cells were double stained with Annexin V–FITC and PI as per manufacturer's instructions (BD Pharmingen). 20,000 events were analyzed on an Epics XL flow cytometer , and percent apoptosis was determined using System II software (Coulter).

### FACS Analysis for hESC Markers

hESCs were grown in six-well plates and submitted to the Human Embryonic Stem Cell Core at Baylor College of Medicine, Houston, Texas, for FACS staining and quality control. Briefly, the cells were removed from the dish with trypsin/EDTA (Invitrogen). Trypsin was neutralized with MEF medium (DMEM containing 10% FBS [Hyclone]) and pelleted by centrifugation for 5 min at 250 *g*. Cells were resuspended in FACS buffer (PBS containing 2% FBS and 0.1% sodium azide) and probed for the surface antigens with SSEA4 (R&D Systems) conjugated with Alexa-488 (Invitrogen). The cells were then fixed with 2% paraformaldehyde for 30 min at RT and permeabilized with 0.1% saponine (Sigma) in PBS with 0.1% BSA (Sigma) for 30 min. Cells were washed with FACS buffer and probed for 30 min at RT with the OCT4 antibody conjugated with R*-*phycoerythrin (both from BD Biosciences) as an intracellular protein. The samples were analyzed using LSRII equipment (BD Biosciences). The cell population of interest was determined and dead cells excluded using forward and side scatter parameters. Acquisition was set for 30,000 events per sample. The data were analyzed with FACSDiva software (version 4.1.2; BD Biosciences). Triplicate samples were analyzed in each experiment.

### Luciferase Reporter Plasmids and Dual Luciferase Assay

Primer pairs were designed to amplify a region of about 200–300 bp around every predicted *miR-34a* target site within the 3′ UTRs of *KLF4* and *LIN28A* (Table S4). The amplicons were cloned in pMir-Report vector (Ambion) at HindIII and SpeI sites. Several residues in the *miR-34a* target site in the 3′ UTRs were mutated using site-directed mutagenesis (Stratagene) with the mutagenesis primers. In the 3′ UTR assay, 10×10^4^ 293 cells were transfected with 100 ng of the UTR reporter (pMir-Report), 10 ng of the transfection control Renilla vector (phRLTK, Promega), and 20 nM pre-*miR-34a* precursor molecules or scrambled control miRNA (Ambion) with 3 µl of Lipofectamine 2000. Twenty-four hours after transfection, cells were lysed in 1× Passive Lysis Buffer, and reporter activity was measured using the Dual Luciferase Assay (Promega). Each assay was tested in triplicate in three independent experiments.

## Supporting Information

Figure S1 **Retinoic acid induces differentiation of hESCs.** (A) WA09 (H9) hESCs were treated with RA for 5 d, and qRT-PCR assay was performed with primers specific for various differentiation markers: *GATA4* and *AFP* (endoderm), *Brachyury* (mesoderm), and *PAX6* and *Nestin* (ectoderm). (B) p53 nuclear localization. Cytosolic and nuclear extracts prepared from hESCs cultured as in (A) were analyzed by Western blotting, blots were quantitated, and average density of three different blots is plotted as relative change in nuclear protein levels. (C) Total cell lysates prepared from mESCs, MEFs, and hESCs were probed with anti-p53 (FL393) antibody. (D and E) WA01 (H1) hESCs were cultured under self-renewing conditions (R0) or treated with RA for 5 d (R1–R5), cells were harvested at indicated time points, p53 protein was analyzed (D), and gene expression was assayed by qRT-PCR (E).
(PDF)

Figure S2 **p53 is activated during differentiation and DNA damage.** (A) hESCs were cultured under self-renewing conditions (R0) or treated with RA for 1 or 2 d, or with DNA-damaging agents Adr (250 ng/ml), or etoposide with trichostatin A for 6 h. Total cell lysates were probed with antibodies against p53, p53K373ac, p53K320ac, p53S15ph, p53S45ph, and actin. (Asterisk indicates non-specific band). (B) Cell lysates from hESCs treated with RA (0–3 d) or Adr (6 h) + MG132 were immunoprecipitated with anti-ubiquitin antibody and probed for p53 to detect ubiquitinated p53. (C) Cell lysates from hESCs treated with RA or Adr (6 h) + lactacystin were immunoprecipitated with anti-p53 antibody and probed for anti-ubiquitin to detect ubiquitinated p53.
(PDF)

Figure S3 **Transfection and knockdown efficiencies of siRNAs in hESCs.** (A and B) Transfection efficiency of siRNA. hESCs were cotransfected with SMARTpools of gene-specific siRNA and siGLO-Green (FAM) and were visualized by microscopy after 24 h (A) or analyzed by flow cytometry to determine the percent of cells transfected with siRNA. (C and D) Knockdown efficiency of siRNA. hESCs transfected twice with siRNA specific to *TP53, HDM2, TRIM24,* and *CDKN1A* were harvested to analyze RNA (C) and protein (D) levels. 36 h after the second transfection we could achieve knockdown efficiency ranging from 70% to 80%. Also see Figure S4.
(PDF)

Figure S4 **The consequence of p53 accumulation in hESCs.** (A–C) hESCs transfected with siRNA and treated with RA (A) or with Adr for 6 h (+Adr) (C) were stained with PI and subjected to flow cytometry analysis. (B) hESCs treated with RA for different times were subjected to cell cycle analysis. (D and E) hESCs exposed to increasing doses of Adr were subjected to Western blot analysis to detect p53 and γ-H2AX (D), and Annexin V staining followed by flow cytometry analysis to determine apoptotic cells (E). Adr at 250 ng/ml concentration was not toxic to hESCs, since only ∼20% hESCs were Annexin V positive (Figure 3E and 3F), whereas the apoptotic response peaked at 1 µg/ml, as shown by stabilization of p53, increased levels of γ-H2AX, and accumulation of apoptotic cells by Annexin V staining. (F) Cell lysates prepared from hESCs treated with Adr or etoposide for 6 h were blotted with anti-RB antibody (left panel). hESCs cultured and treated as in Figure 1 were lysed, and total cell lysates were analyzed by Western blotting (right panel). (G) qRT-PCR assay. hESCs cultured under differentiation conditions (+RA 0–4 d) or treated with Adr for 6 h were subjected to qRT-PCR assay using primers specific for human *BAX, GADD45A*, and normalized to *Actin*. (H) ChIP. p53-bound chromatin was immunoprecipitated from hESCs treated with Adr, and p53 enrichment on *CDKN1A, HDM2, BAX*, and *GADD45A* was analyzed by qRT-PCR using primers encompassing p53REs and plotted as fold p53 enrichment compared to input.
(PDF)

Figure S5 **p53 regulates differentiation of hESCs.** (A) Western blotting. hESCs cultured under self-renewing conditions were transfected with either non-target (siControl) or siRNAs specific to *HDM2 (siHDM2), TRIM24 (siTRIM24)*, or *CDKN1A (siCDKN1A)*. 36 h post-transfection cells were cultured in complete (R0) or in differentiating medium for 3 d (R1, R2, and R3). Total cell lysates were analyzed as indicated. Western blots were quantitated, and average density of three different blots is plotted as fold change in protein levels. (B) RNA analysis. Cells treated as in (A) were harvested and subjected to gene expression analysis by qRT-PCR.
(PDF)

Figure S6 **DNA binding activity of p53 is required to induce differentiation of hESCs.** (A) Quality control of hESCs stably expressing tet-inducible p53. WA09 p53WT, WA09 p53R175P and WA09 p53R175H cells were fixed in formaldehyde and stained with ESC surface marker SSEA4 and ESC internal marker OCT4. hESCs were subjected to dual flow cytometry analysis using LSRII equipment (BD Biosciences). The cell population of interest was determined and dead cells excluded using forward and side scatter parameters. Acquisition was set for 30,000 events per sample. The data were analyzed with FACSDiva software (version 4.1.2). Triplicate samples were analyzed in each experiment. (B and C) qRT-PCR assay. RNA prepared from cells expressing exogenous Dox-inducible p53 were analyzed for mRNA levels of endogenous *TP53* (left panel), *HDM2*, *NANOG* and, *KLF4* by qRT-PCR assay. Data are presented as mean ± standard error of the mean (SEM).
(PDF)

Figure S7 **p53 transcriptionally regulates miRNAs in hESCs.** (A) miRNA-TaqMan assay. WA09 cells cultured under self-renewing conditions (control) or treated with Adr were lysed to prepare RNA, TaqMan qRT-PCR assay was performed with probes specific for human *miR-34a* and *miR-145* and were normalized to RNU6B. (B) ChIP. Chromatin was immunoprecipitated using p53 antibody from WA09 cells treated with Adr. p53 binding was analyzed by qRT-PCR on *miR-34a* and *miR-145* promoters using primers encompassing p53REs. Primers amplifying nonspecific promoter regions were used as negative control. (C) Knockdown efficiency of miRNAs. hESCs transfected with anti-miRNA specific to *miR-34a* and *miR-145* were harvested to analyze miRNA levels by TaqMan assay in (A). (D) Cell cycle analysis. hESCs transfected with either anti-*miR-NS* (control) or anti-*miR-145* oligonucleotides and treated with RA were stained with PI and subjected to flow cytometry analysis. (E) List of targets of *miR-34a* and *miR-145* that are significant to p53 and hESC biology identified by TargetScan, PicTar, miRanda, and miRBase browsers (asterisks indicate validated targets).
(PDF)

Table S1 **Sequence information for the siGENOME SMARTpool (Dharmacon) siRNAs.**
(DOC)

Table S2 **Oligonucleotide sequences for gene expression analysis by qRT-PCR.**
(DOC)

Table S3 **Oligonucleotide sequences for qRT-PCR analysis after p53 ChIP.**
(DOC)

Table S4 **Oligonucleotide sequences for cloning 3′ UTRs in pMir-Report luciferase vector.**
(DOC)

## Abbreviations

Adr: adriamycin
AP: alkaline phosphatase
ChIP: chromatin immunoprecipitation
Dox: doxycycline
CM: conditioned medium
ESC: embryonic stem cell
FGF: fibroblast growth factor
hESC: human embryonic stem cell
iPSC: induced pluripotent stem cell
MEF: mouse embryonic fibroblast
mESC: mouse embryonic stem cell
miRNA: microRNA
PI: propidium iodide
p53RE: p53 response element
p53WT: wild-type p53
qRT-PCR: quantitative real-time PCR
RA: retinoic acid
RT: room temperature
SDS-PAGE: sodium dodecyl sulfate polyacrylamide gel electrophoresis
SEM: standard error of the mean
siRNA: small interfering RNA
