# Supplementary figures and images for: p53 Regulates Cell Cycle and MicroRNAs to Promote Differentiation of Human Embryonic Stem Cells
Source: PLoS Biol. 2012 Feb 28;10(2):e1001268. doi: 10.1371/journal.pbio.1001268 (PMC3289600; doi:10.1371/journal.pbio.1001268)

**A**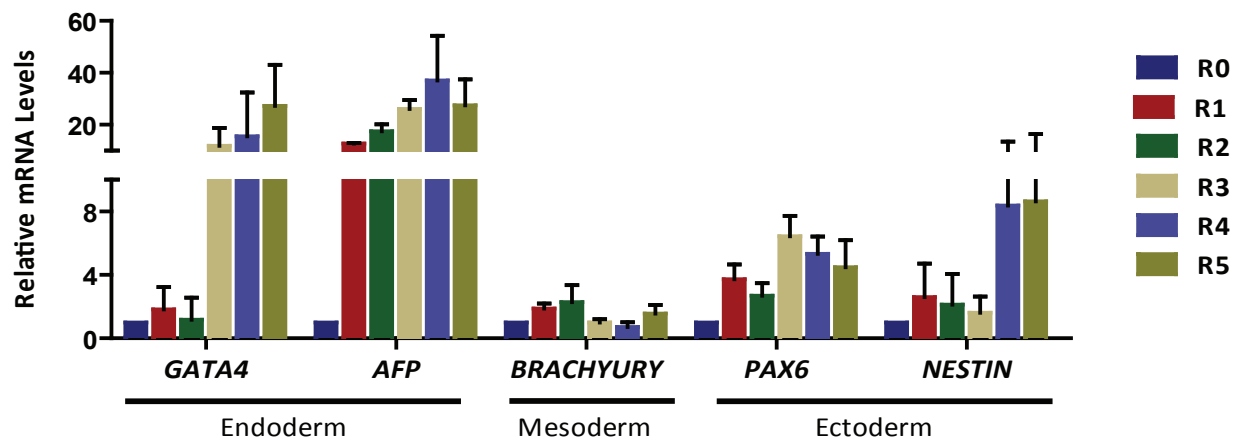**B**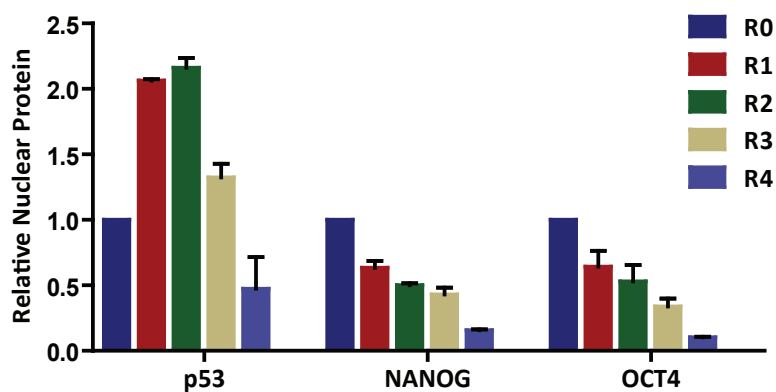**C**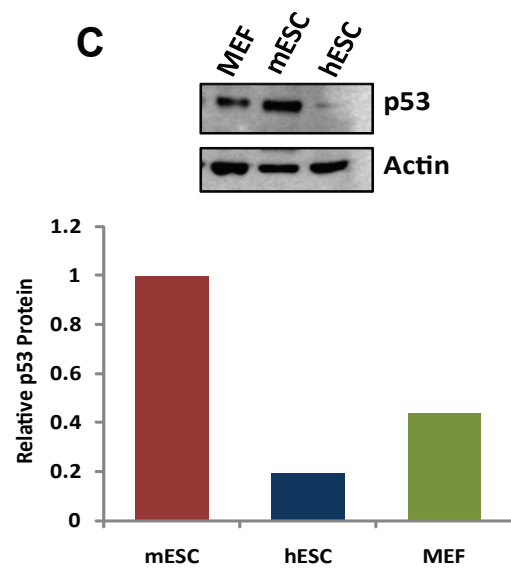**D**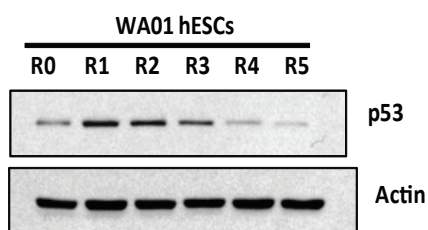**E**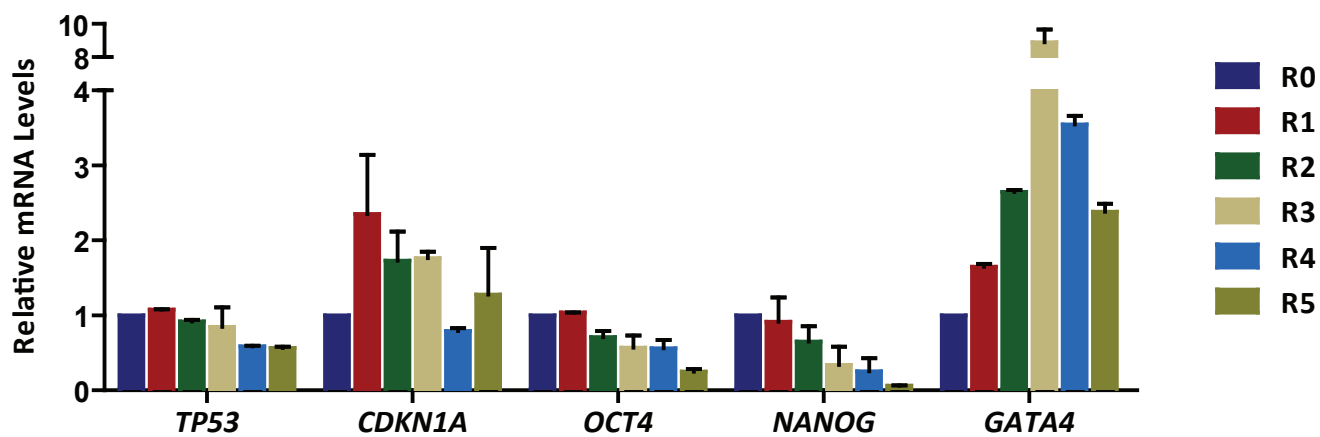

Supplement: Figure S1 — Retinoic acid induces differentiation of hESCs. (A) WA09 (H9) hESCs were treated with RA for 5 d, and qRT-PCR assay was performed with primers specific for various differentiation markers: GATA4 and AFP (endoderm), Brachyury (mesoderm), and PAX6 and Nestin (ectoderm). (B) p53 nuclear localization. Cytosolic and nuclear extracts prepared from hESCs cultured as in (A) were analyzed by Western blotting, blots were quantitated, and average density of three different blots is plotted as relative change in nuclear protein levels. (C) Total cell lysates prepared from mESCs, MEFs, and hESCs were probed with anti-p53 (FL393) antibody. (D and E) WA01 (H1) hESCs were cultured under self-renewing conditions (R0) or treated with RA for 5 d (R1–R5), cells were harvested at indicated time points, p53 protein was analyzed (D), and gene expression was assayed by qRT-PCR (E). (PDF) [file pbio.1001268.s001.pdf]

**A**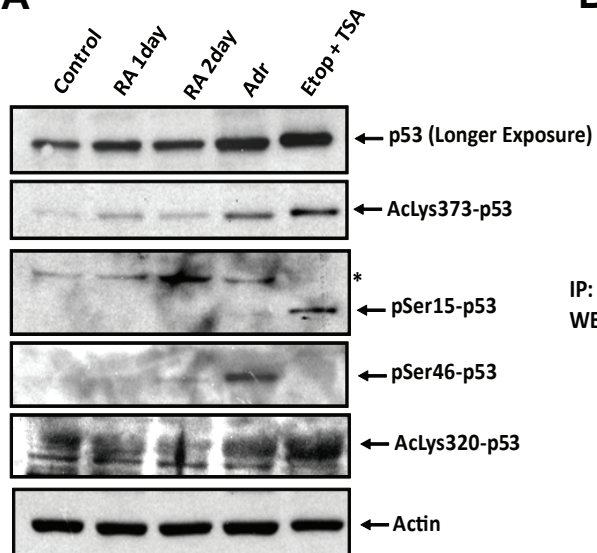**B**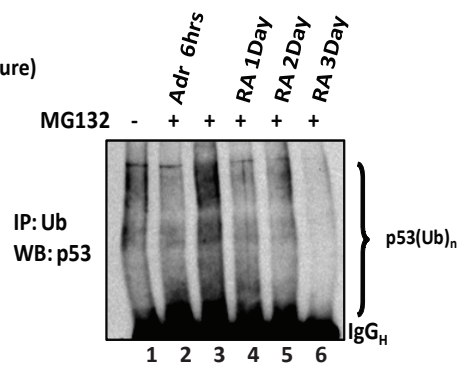**C**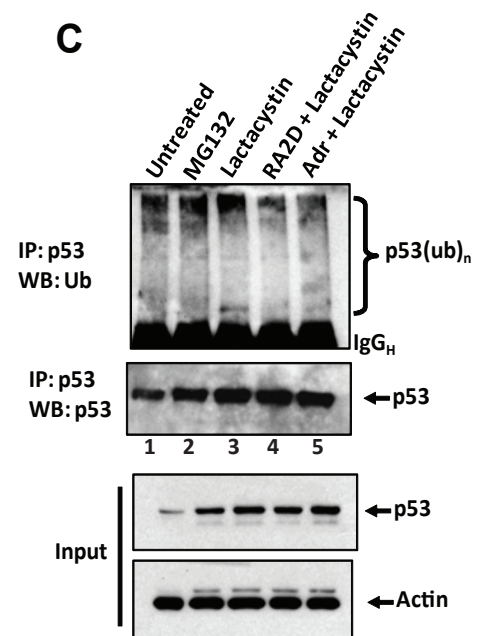

Supplement: Figure S2 — p53 is activated during differentiation and DNA damage. (A) hESCs were cultured under self-renewing conditions (R0) or treated with RA for 1 or 2 d, or with DNA-damaging agents Adr (250 ng/ml), or etoposide with trichostatin A for 6 h. Total cell lysates were probed with antibodies against p53, p53K373ac, p53K320ac, p53S15ph, p53S45ph, and actin. (Asterisk indicates non-specific band). (B) Cell lysates from hESCs treated with RA (0–3 d) or Adr (6 h) + MG132 were immunoprecipitated with anti-ubiquitin antibody and probed for p53 to detect ubiquitinated p53. (C) Cell lysates from hESCs treated with RA or Adr (6 h) + lactacystin were immunoprecipitated with anti-p53 antibody and probed for anti-ubiquitin to detect ubiquitinated p53. (PDF) [file pbio.1001268.s002.pdf]

**A**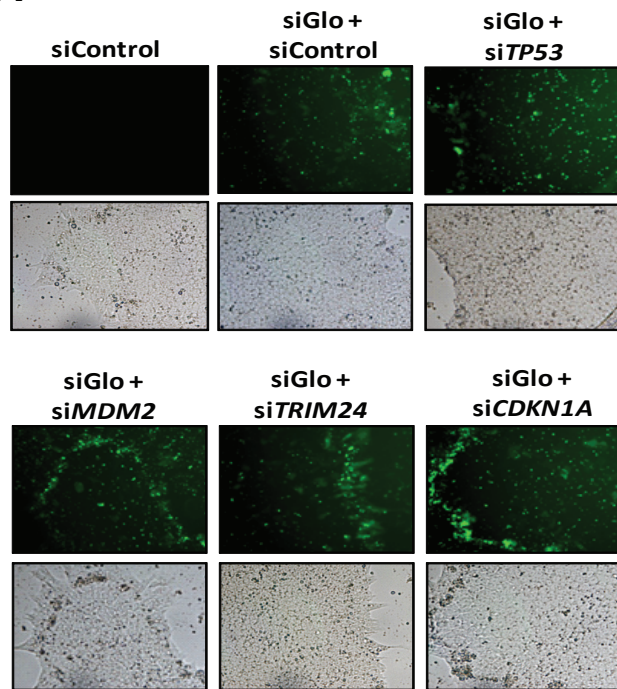**B**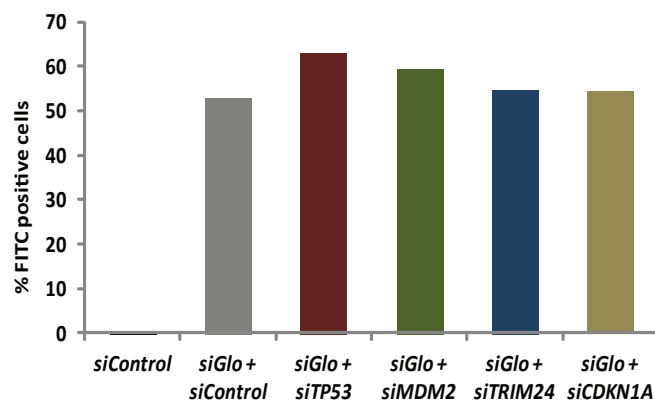**C**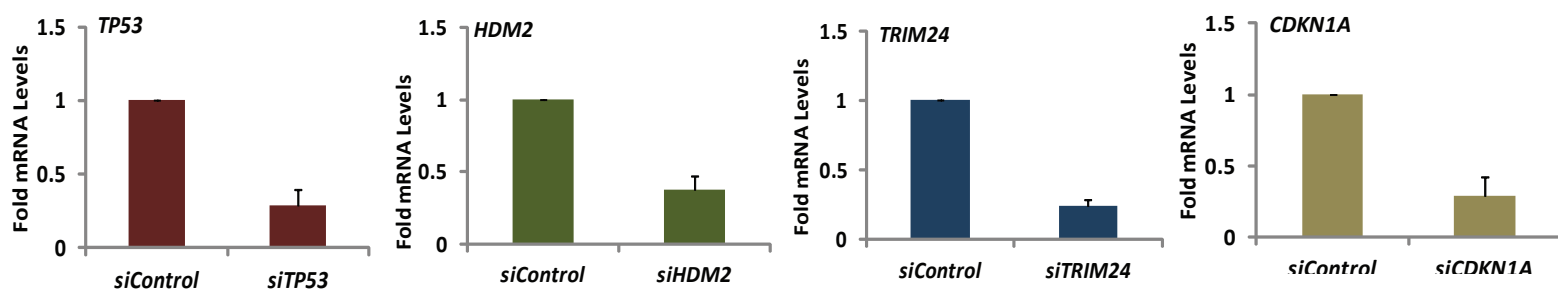**D**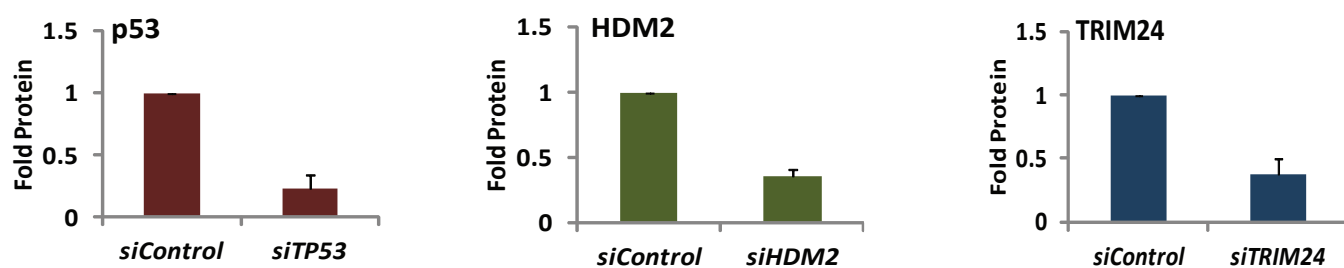

Supplement: Figure S3 — Transfection and knockdown efficiencies of siRNAs in hESCs. (A and B) Transfection efficiency of siRNA. hESCs were cotransfected with SMARTpools of gene-specific siRNA and siGLO-Green (FAM) and were visualized by microscopy after 24 h (A) or analyzed by flow cytometry to determine the percent of cells transfected with siRNA. (C and D) Knockdown efficiency of siRNA. hESCs transfected twice with siRNA specific to TP53, HDM2, TRIM24, and CDKN1A were harvested to analyze RNA (C) and protein (D) levels. 36 h after the second transfection we could achieve knockdown efficiency ranging from 70% to 80%. Also see Figure S4. (PDF) [file pbio.1001268.s003.pdf]

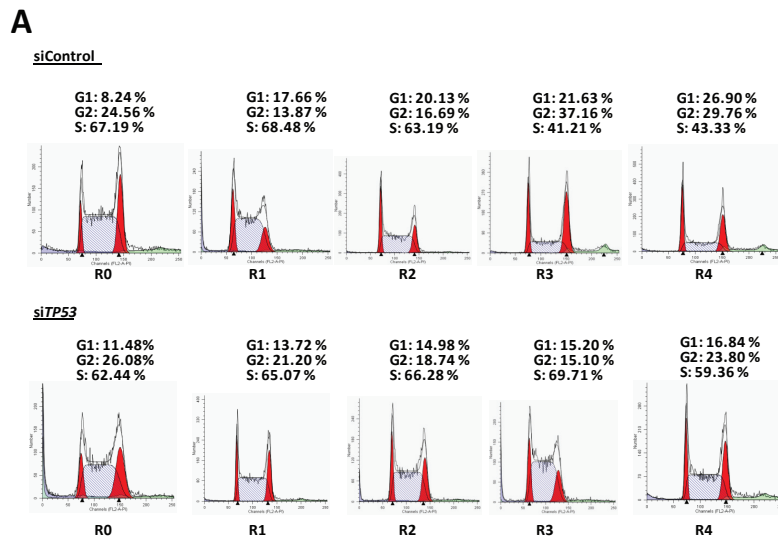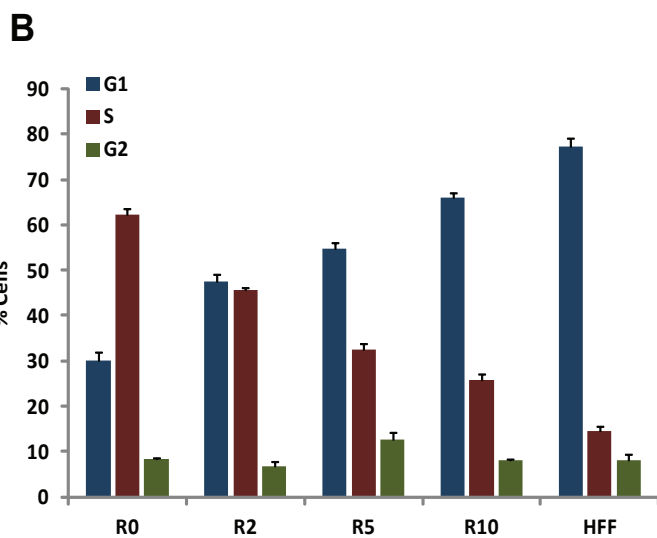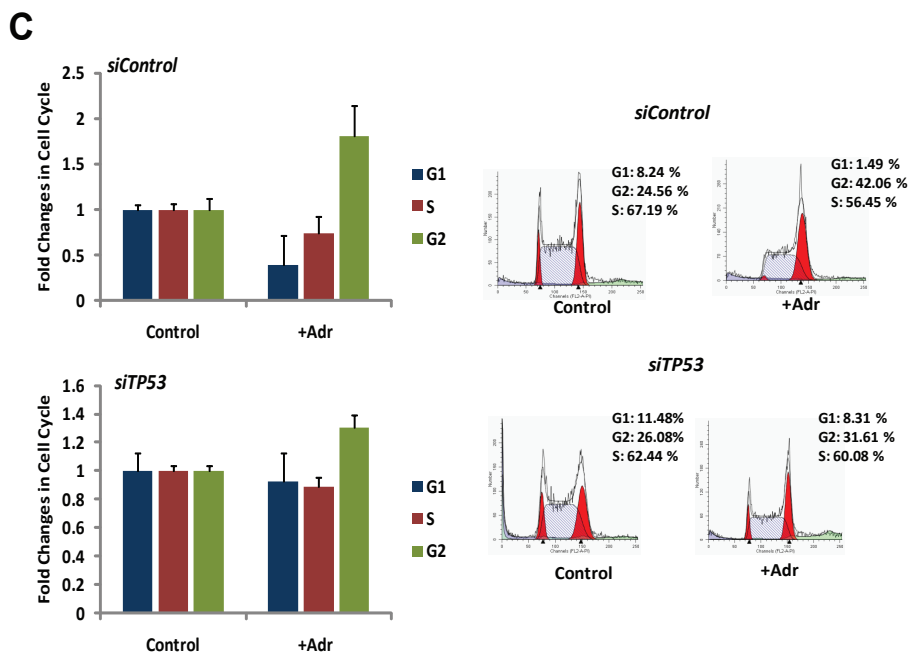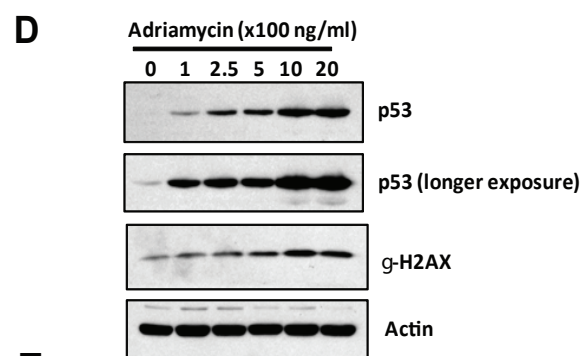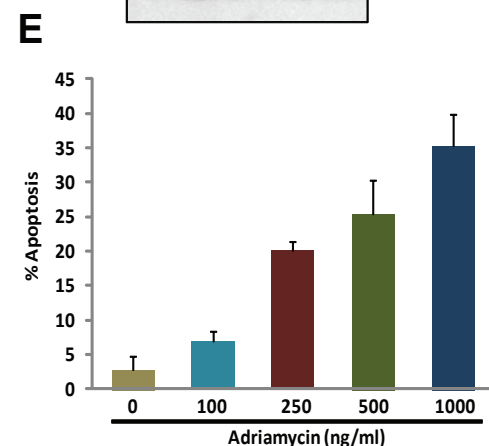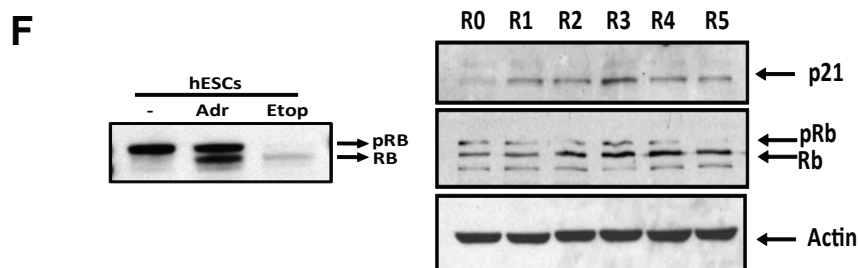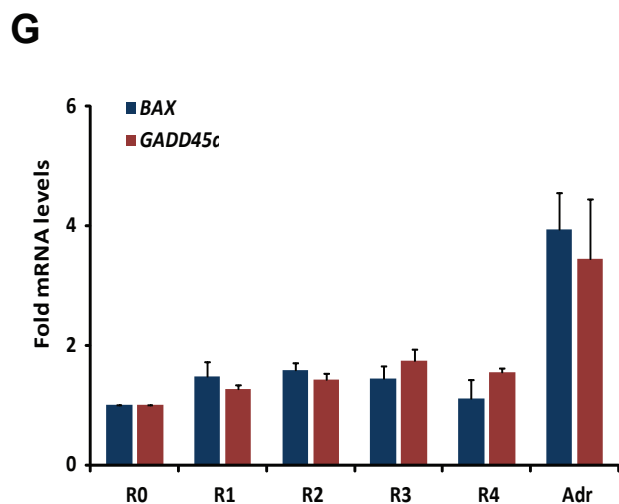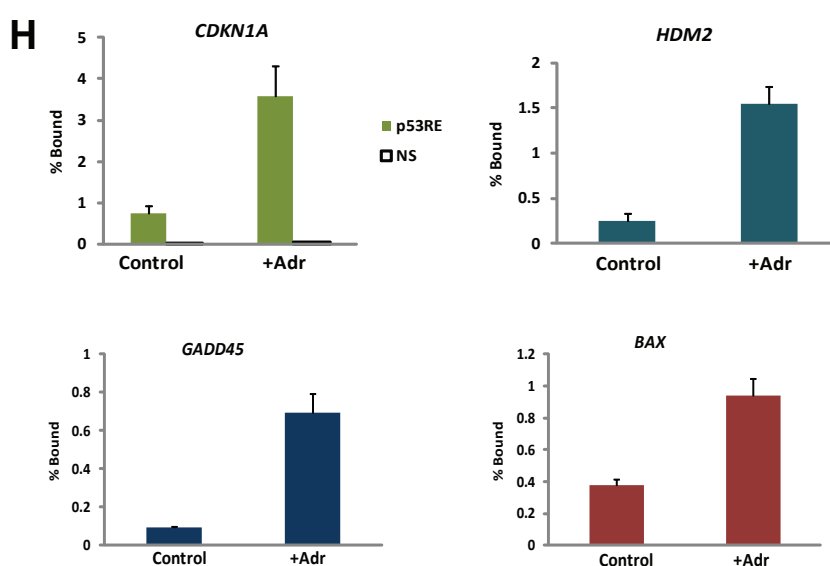

Supplement: Figure S4 — The consequence of p53 accumulation in hESCs. (A–C) hESCs transfected with siRNA and treated with RA (A) or with Adr for 6 h (+Adr) (C) were stained with PI and subjected to flow cytometry analysis. (B) hESCs treated with RA for different times were subjected to cell cycle analysis. (D and E) hESCs exposed to increasing doses of Adr were subjected to Western blot analysis to detect p53 and γ-H2AX (D), and Annexin V staining followed by flow cytometry analysis to determine apoptotic cells (E). Adr at 250 ng/ml concentration was not toxic to hESCs, since only ∼20% hESCs were Annexin V positive (Figure 3E and 3F), whereas the apoptotic response peaked at 1 µg/ml, as shown by stabilization of p53, increased levels of γ-H2AX, and accumulation of apoptotic cells by Annexin V staining. (F) Cell lysates prepared from hESCs treated with Adr or etoposide for 6 h were blotted with anti-RB antibody (left panel). hESCs cultured and treated as in Figure 1 were lysed, and total cell lysates were analyzed by Western blotting (right panel). (G) qRT-PCR assay. hESCs cultured under differentiation conditions (+RA 0–4 d) or treated with Adr for 6 h were subjected to qRT-PCR assay using primers specific for human BAX, GADD45A, and normalized to Actin. (H) ChIP. p53-bound chromatin was immunoprecipitated from hESCs treated with Adr, and p53 enrichment on CDKN1A, HDM2, BAX, and GADD45A was analyzed by qRT-PCR using primers encompassing p53REs and plotted as fold p53 enrichment compared to input. (PDF) [file pbio.1001268.s004.pdf]

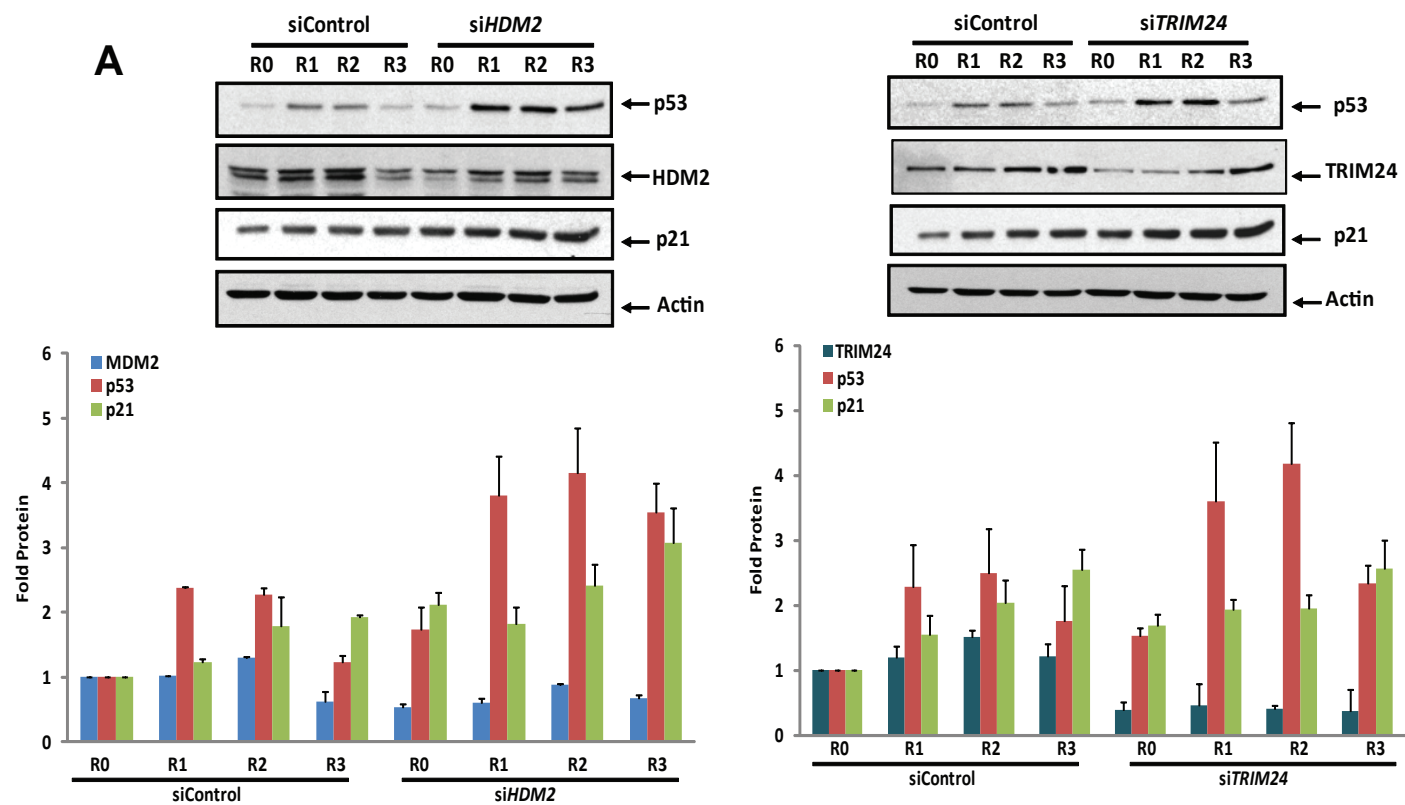

**B**

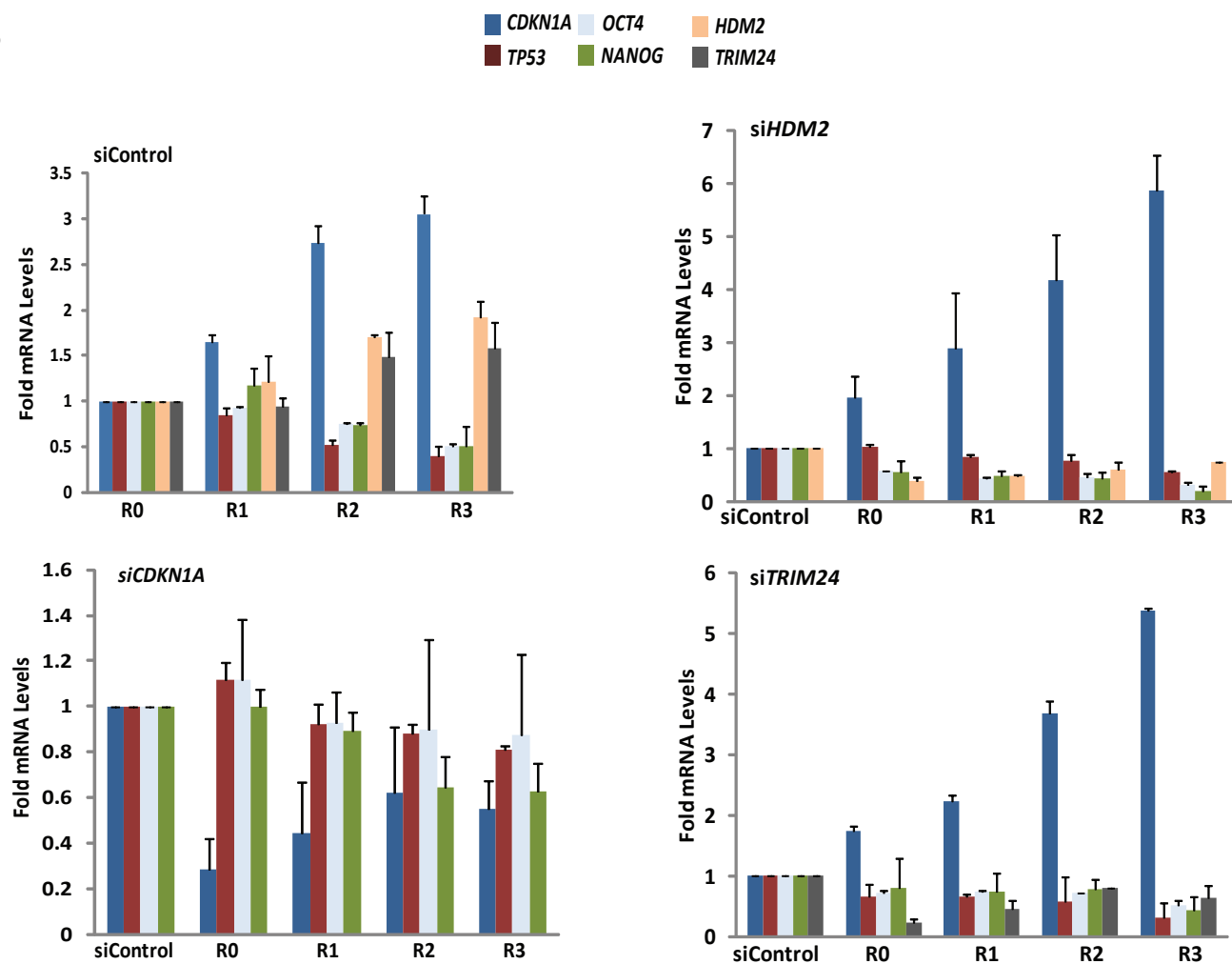

Supplement: Figure S5 — p53 regulates differentiation of hESCs. (A) Western blotting. hESCs cultured under self-renewing conditions were transfected with either non-target (siControl) or siRNAs specific to HDM2 (siHDM2), TRIM24 (siTRIM24), or CDKN1A (siCDKN1A). 36 h post-transfection cells were cultured in complete (R0) or in differentiating medium for 3 d (R1, R2, and R3). Total cell lysates were analyzed as indicated. Western blots were quantitated, and average density of three different blots is plotted as fold change in protein levels. (B) RNA analysis. Cells treated as in (A) were harvested and subjected to gene expression analysis by qRT-PCR. (PDF) [file pbio.1001268.s005.pdf]

**A**

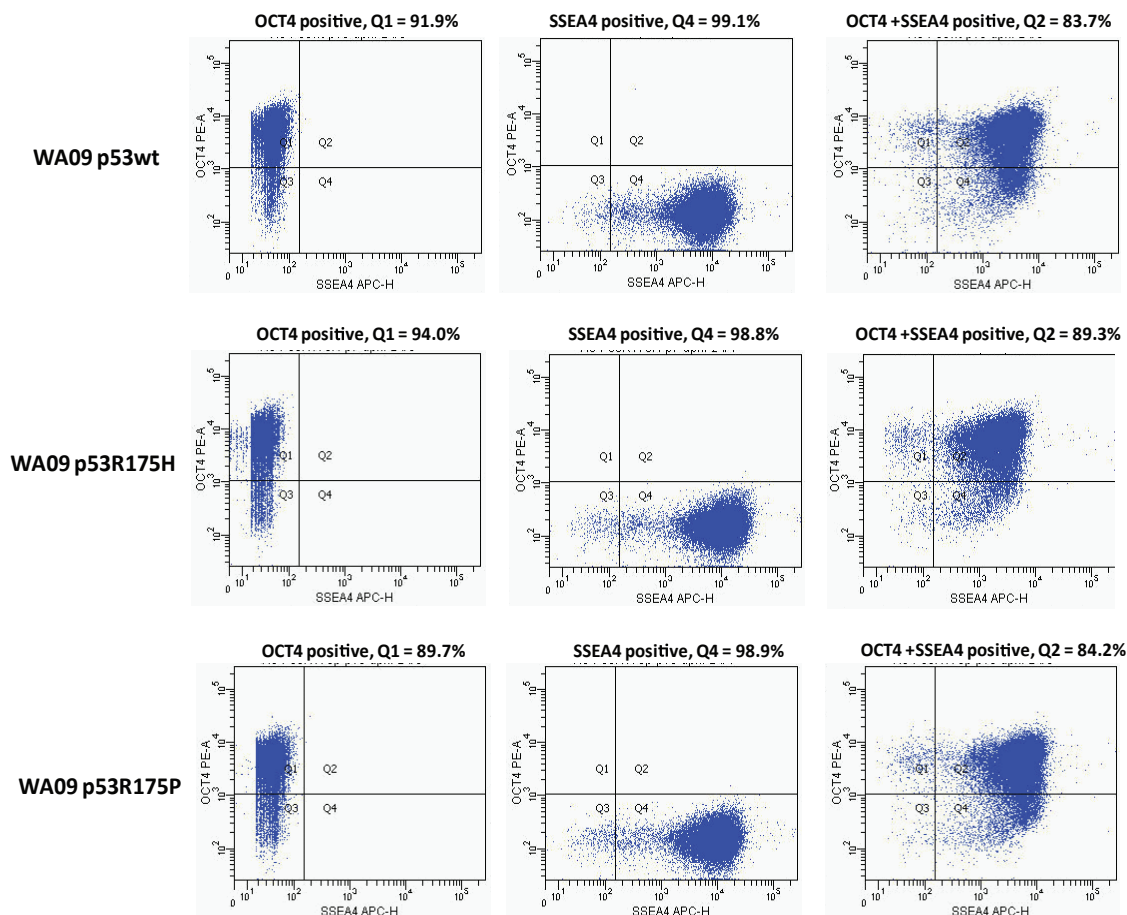

**B**

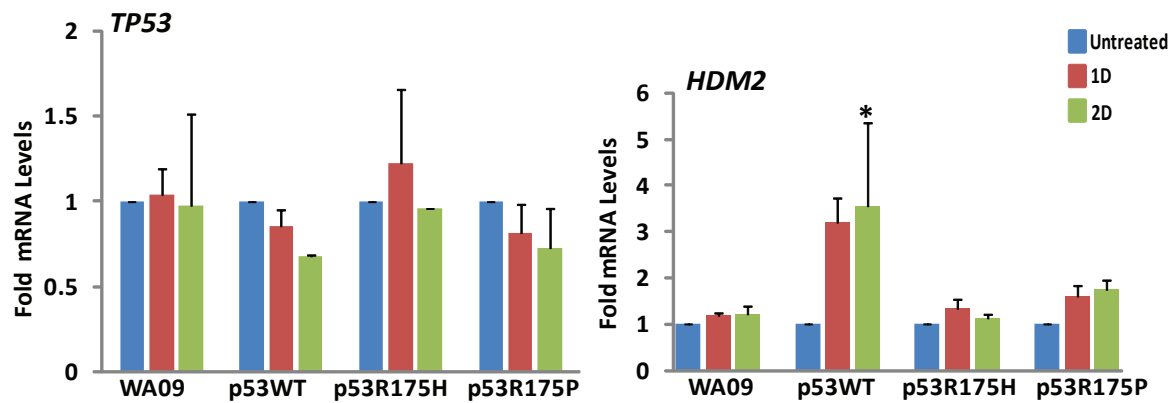

**C**

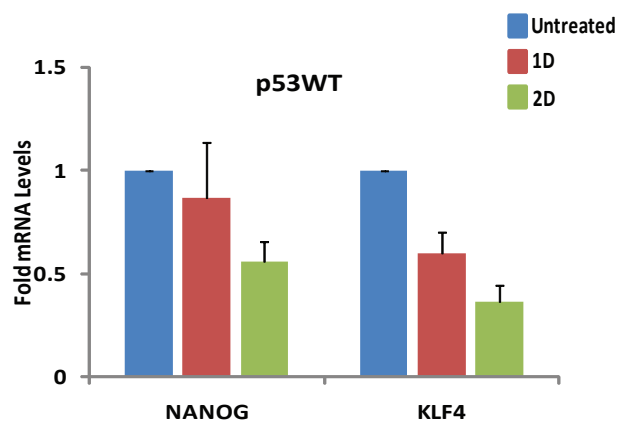

Supplement: Figure S6 — DNA binding activity of p53 is required to induce differentiation of hESCs. (A) Quality control of hESCs stably expressing tet-inducible p53. WA09 p53WT, WA09 p53R175P and WA09 p53R175H cells were fixed in formaldehyde and stained with ESC surface marker SSEA4 and ESC internal marker OCT4. hESCs were subjected to dual flow cytometry analysis using LSRII equipment (BD Biosciences). The cell population of interest was determined and dead cells excluded using forward and side scatter parameters. Acquisition was set for 30,000 events per sample. The data were analyzed with FACSDiva software (version 4.1.2). Triplicate samples were analyzed in each experiment. (B and C) qRT-PCR assay. RNA prepared from cells expressing exogenous Dox-inducible p53 were analyzed for mRNA levels of endogenous TP53 (left panel), HDM2, NANOG and, KLF4 by qRT-PCR assay. Data are presented as mean ± standard error of the mean (SEM). (PDF) [file pbio.1001268.s006.pdf]

**A**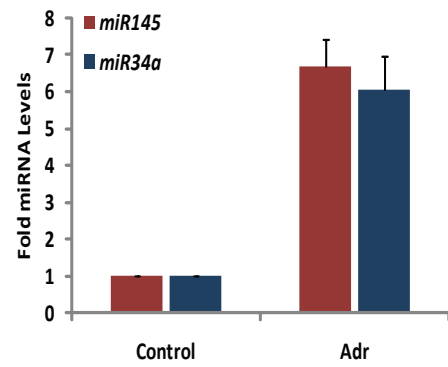**B**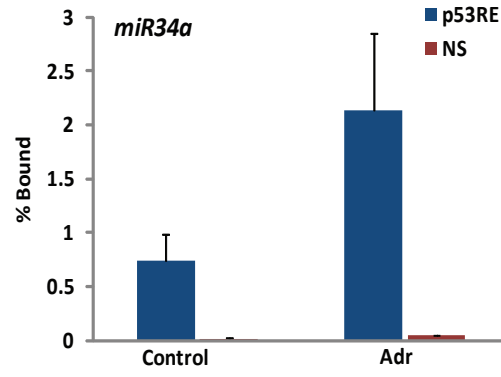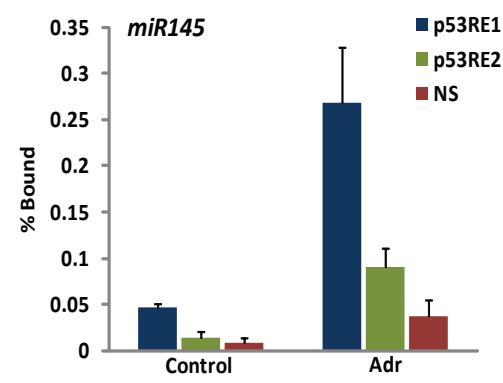**C**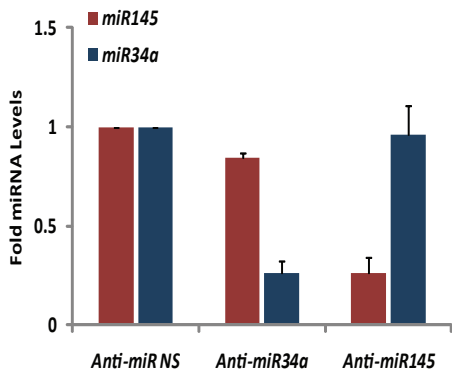**D**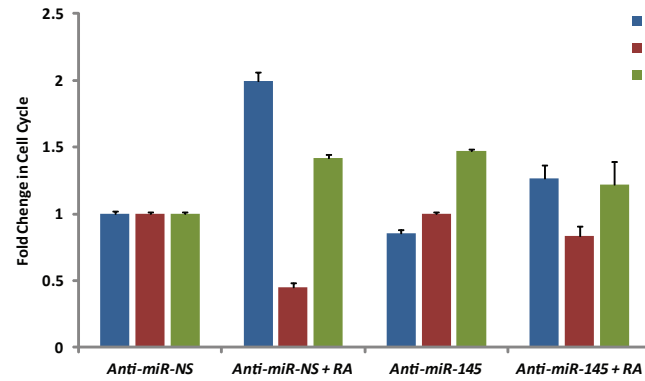**E**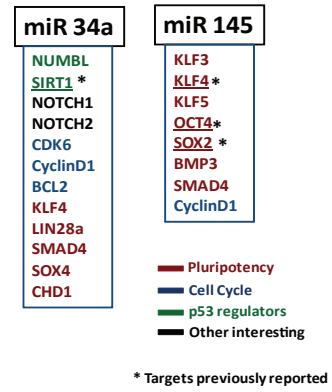

Supplement: Figure S7 — p53 transcriptionally regulates miRNAs in hESCs. (A) miRNA-TaqMan assay. WA09 cells cultured under self-renewing conditions (control) or treated with Adr were lysed to prepare RNA, TaqMan qRT-PCR assay was performed with probes specific for human miR-34a and miR-145 and were normalized to RNU6B. (B) ChIP. Chromatin was immunoprecipitated using p53 antibody from WA09 cells treated with Adr. p53 binding was analyzed by qRT-PCR on miR-34a and miR-145 promoters using primers encompassing p53REs. Primers amplifying nonspecific promoter regions were used as negative control. (C) Knockdown efficiency of miRNAs. hESCs transfected with anti-miRNA specific to miR-34a and miR-145 were harvested to analyze miRNA levels by TaqMan assay in (A). (D) Cell cycle analysis. hESCs transfected with either anti-miR-NS (control) or anti-miR-145 oligonucleotides and treated with RA were stained with PI and subjected to flow cytometry analysis. (E) List of targets of miR-34a and miR-145 that are significant to p53 and hESC biology identified by TargetScan, PicTar, miRanda, and miRBase browsers (asterisks indicate validated targets). (PDF) [file pbio.1001268.s007.pdf]
