# Supplementary material for: p53 Regulates Cell Cycle and MicroRNAs to Promote Differentiation of Human Embryonic Stem Cells
Source: PLoS Biol. 2012 Feb 28;10(2):e1001268. doi: 10.1371/journal.pbio.1001268 (PMC3289600; doi:10.1371/journal.pbio.1001268)
Supplement: Table S1 — Sequence information for the siGENOME SMARTpool (Dharmacon) siRNAs. (DOC) [file pbio.1001268.s008.doc]

**Supplementary Table S1.** Sequence information for the siGENOME SMARTpool (Dharmacon) siRNAs

| **Target Gene** | **Catalog #** | **siRNA Sequences** |
| --- | --- | --- |
| Human *TP53* | M-003329-03-0005 | GAGGUUGGCUCUGACUGUA  GCACAGAGGAAGAGAAUCU  GAAGAAACCACUGGAUGGA  GCUUCGAGAUGUUCCGAGA |
| Human *MDM2* | M-003279-04-0005 | GCCAGUAUAUUAUGACUAA  GAUGAGAAGCAACAACAUA  AAAGUCUGUUGGUGCACAA  CCCUAGGAAUUUAGACAAC |
| Human *TRIM24* | M-005387-03-0010 | CUUUAUAGCAAACGACUGA  CUUUAGUAAUCGAGGAUAA  AGACUUAUCUAAACCAGAA  GAACAUACCACGACAAGCA |
| Human *CDKN1A* | M-003471-00-0005 | GAUGGAACUUCGACUUUGU  GCGAUGGAACUUCGACUUU  CGAUGGAACUUCGACUUUG  CGACUGUGAUGCGCUAAUG |
