# Supplementary material for: p53 Regulates Cell Cycle and MicroRNAs to Promote Differentiation of Human Embryonic Stem Cells
Source: PLoS Biol. 2012 Feb 28;10(2):e1001268. doi: 10.1371/journal.pbio.1001268 (PMC3289600; doi:10.1371/journal.pbio.1001268)
Supplement: Table S2 — Oligonucleotide sequences for gene expression analysis by qRT-PCR. (DOC) [file pbio.1001268.s009.doc]

**Supplementary Table S2.** Oligonucleotide sequences for gene expression analysis by Real Time qRT-PCR

| **Gene** | **Primers** |
| --- | --- |
| hOCT4-5' | ACATCAAAGCTCTGCAGAAAGAACT |
| hOCT4-3' | CTGAATACCTTCCCAAATAGAACCC |
| hNANOG-5’ | CCGAAGAATAGCAATGGTGTGACG |
| hNANOG-3’ | AGGAGAATTTGGCTGGAACTGC |
| hSOX2-5' | CGCCGCCCCCAGCAGACTTCACAT |
| hSOX2-3' | TGCACCCCTCCCATTTCCCTCGTT |
| hKLF4-5' | CCGCTCCATTACCAAGAGCT |
| hKLF4-3' | CTCCTCTGGCATGCAGGAA |
| hAFP-5' | CGAGGGAGCGGCTGACATTATTAT |
| hAFP-3' | TGGCCTTGGCAGCATTTCTCC |
| hGATA4-5' | CAATCTCGATATGTTTGACGACTT |
| hGATA4-3' | TGCCGTTCATCTTGTGGTAGAG |
| hBRACHYURY-5' | GCAAAGGAAAGAAGTGATCACAAA |
| hBRACHYURY-3' | GAAGAAGCCACCCCCATTGG |
| hPAX6-5' | CGGAAGCTGCAAAGAAATAGAAC |
| hPAX6-3' | AACTCTTTCTCCAGGGCCTCAA |
| hNestin-5’ | GAGAGCCCTGAGCCCAAAGA |
| hNestin-3’ | CTCCCGCAGCAGACTCACC |
| hTP53-5' (3’-UTR) | TGCAATAGGTGTGCGTCAGAA |
| hTP53-3' (3’-UTR) | CCCCGGGACAAAGCAAA |
| hTP53-5’(Coding region) | GCGAGCACTGCCCAACAACA |
| hTP53-3’(Coding region) | GGATCTGAAGGGTGAAATATTCT |
| hCDKN1A-5' | TACCCTTGTGCCTCGCTCAG |
| hCDKN1A-3' | CGGCGTTTGGAGTGGTAGA |
| hHDM2-5' | CTGGCTCTGTGTGTAATAAGGGAG |
| hHDM2-3' | CCTGATCCAACCAATCACCTG |
| hACTIN-5' | GTGGATCAGCAAGCAGGAG |
| hACTIN-3' | TTTGTCAAGAAAGGGTGTAACG |
