# Supplementary material for: p53 Regulates Cell Cycle and MicroRNAs to Promote Differentiation of Human Embryonic Stem Cells
Source: PLoS Biol. 2012 Feb 28;10(2):e1001268. doi: 10.1371/journal.pbio.1001268 (PMC3289600; doi:10.1371/journal.pbio.1001268)
Supplement: Table S3 — Oligonucleotide sequences for qRT-PCR analysis after p53 ChIP. (DOC) [file pbio.1001268.s010.doc]

**Supplementary Table S3.** Oligonucleotide sequences for Real Time qPCR analysis after p53-ChIP

| **p53RE on Gene** | **Primers** |
| --- | --- |
| hCDKN1Ap53R-5' | AGCAGGCTGTGGCTCTGATT |
| hCDKN1Ap53RE-3' | CAAAATAGCCACCAGCCTCTTCT |
| hBAXp53RE1-5' | CGCTCTCGGACCCTCGAGAA |
| hBAXp53RE1-3' | TGCATCCAGACCCCTGGCCT |
| hGADD45p53RE-5' | TGTGGTACAGAACATGTCTAAGC |
| hGADD45p53RE-3' | TGCAGATGTAGGTAGGGAGTAG |
| hNANOG NS-5' | AGCCTCAACCCTGAGATCAAG |
| hNANOG NS-3' | TAGCCATGCAGAGTGGGTCAG |
| hmiR-34ap53RE-5' | ATGCCCCCGATCTGCGTGGT |
| hmiR-34ap53RE-3' | TGGTGGCACGAGCAGGAAGGA |
| hmiR-34a NS-5' | GGAGAGGGCGTACTCAAACC |
| hmiR-34a NS-3' | TGGAGTAAAATGCCTTGAGTTG |
| hmiR-145p53RE1-5' | CTTCACGAGTCTGGGCATGT |
| hmiR-145p53RE1-3' | CCATGGGCTCAGAAAGAGAA |
| hmiR-145p53RE2-5' | AGGGTCTTCACTCTGTACTTCTA |
| hmiR-145p53RE2-3' | CCTCTGCTGCTCATTGTGCTT |
| hmiR-145 NS-5' | TTCAACTCCTCTGGTCCAAGCT |
| hmiR-145 NS-3' | GCTGCTGGCAAGGGAATGTTA |
