# Supplementary material for: p53 Regulates Cell Cycle and MicroRNAs to Promote Differentiation of Human Embryonic Stem Cells
Source: PLoS Biol. 2012 Feb 28;10(2):e1001268. doi: 10.1371/journal.pbio.1001268 (PMC3289600; doi:10.1371/journal.pbio.1001268)
Supplement: Table S4 — Oligonucleotide sequences for cloning 3′ UTRs in pMir-Report luciferase vector. (DOC) [file pbio.1001268.s011.doc]

**Supplementary Table S4.** Oligonucleotide sequences for cloning 3’UTRs in pMir-Report Luciferase vector.

| **Gene** | **Primers** |
| --- | --- |
| hKLF4 3’UTR-WT-5’ | CTAG AAGCTT CTCACCTTGAGTATG |
| hKLF4 3’UTR-WT-3’ | CTAG ACTAGT ATCCCAGACAGTGGA |
| hKLF4 3’UTR-MUT-5’ | GGATATGACCC**TCAGTCCG**AGAAGAGAATTCAG |
| hKLF4 3’UTR-MUT-3’ | CTGAATTCTCTTCTCGGACTGAGGGTCATATCC |
| hLIN28A 3’UTR-WT-5’ | CTAG AAGCTT GGCTCCATGAATCTG |
| hLIN28A 3’UTR-WT-3’ | CTAG ACTAGT GAATTGAGCCACA |
| hLIN28A 3’UTR-MUT-5’ | GGTGCATTGGGG**C**T**AG**T**TC**G**CAGTCCG**ATGTATCTC |
| hLIN28A 3’UTR-MUT-3’ | GAGATACATCGGACTGCGAACTAGCCCCAATGCACC |
